# Probabilistic analysis of water-sealed performance in underground oil storage considering spatial variability of hydraulic conductivity

**DOI:** 10.1038/s41598-022-16960-3

**Published:** 2022-08-12

**Authors:** Huijie Zhang, Bin Zhang, Yajun Li, Lei Wang, Yutao Li, Lei Shi, Hanxun Wang

**Affiliations:** 1 School of Engineering and Technology, China University of Geosciences (Beijing), Beijing, 100083 People’s Republic of China; 2 Department of Civil Engineering, University of the District of Columbia, Washington, DC 20008 USA

**Keywords:** Energy science and technology, Engineering

## Abstract

For underground water-sealed oil storage, the spatial variability of the surrounding rock has a significant impact on the water-sealed effect of a water curtain system. This study presents a methodology for the probabilistic analysis of water curtain performance in underground oil storage, considering the spatial variability of hydraulic conductivity of the surrounding rock based on field data. Anisotropic random fields representing the spatial variability of hydraulic conductivity were established through spatial statistical analysis of field data and introduced into the finite element model of underground oil storage for water-sealed reliability analysis. The water-sealed performance of different water curtain system schemes was studied using Monte Carlo simulation (MCS). The results showed that the difference between the horizontal spatial correlation and the vertical spatial correlation of the surrounding rock has a significant impact on the water-sealed effect of the water curtain system. An excessively large pressure of water curtain boreholes provided a small contribution to improving water curtain performance. The distance between the water curtain holes and the caverns had the less significant affecting the water-sealed reliability of the storage cavern. Finally, the optimal design of the water curtain system is discussed. This study provides valuable insights and a theoretical basis for the optimisation of water curtain system design parameters for underground water-sealed oil storage.

## Introduction

Underground water-sealed oil storage has proven to be a cost-effective approach for maintaining strategic petroleum reserves worldwide because of its improved safety, geographical adaptability, and lowmaintenance cost^1,2^. In order to store crude oil in storage caverns and to ensure the containment reliability, the hydraulic potential in surrounding rock should be higher than the storage caverns potential^3^. For the purpose of maintaining a stable groundwater level, Professor Ingvar Janelid proposed a method using artificial water curtain system^4^. Underground water-sealed oil storage units are generally constructed in areas with relatively intact rock formations^5^. However, a rock mass exhibits spatial variability owing to spatial nonuniformity and fracture development. The spatial variability of the surrounding rock (especially hydraulic conductivity) has a considerable influence on the water-sealed effect of the water curtain system in underground oil storage. The water curtain system plays a vital role in the safe operation of underground water-sealed oil storage and protection of groundwater resources. Performance of the water curtain system has become the main focus in the safety assessment of underground water-sealed oil storage^6,7^.

At present, studies on water curtain system performance in underground oil storage focus on physical model experiments and numerical simulation methods. Rehbinder et al.^8^ combined theoretical analysis and physical model experiments to explore the relationships between the water curtain borehole spacing and the pressures in the cavity. Li et al.^9^ developed an experimental physical modelling system to evaluate the performance of water curtain system with different geometrical parameters. The experiments indicated that the performance of water curtain system is strongly influenced by the water curtain borehole spacing. However, physical model experiments are customised for specific conditions, and the results may not be universal. At present, numerical simulation methods have been widely developed and adapted to study the water-sealed effect and to optimise the water curtain system. Ravandi et al.^10^ conducted a sensitivity analysis on the effect of water curtain parameters. The result showed that the pressure of water curtain boreholes and the distance between water curtain boreholes and caverns have significant influence on the performance of water curtain system. Xu et al.^11^ analysed the groundwater seepage field around caverns and water inflow with different water curtain parameters by numerical simulation. The effects of these parameters on the performance of water curtain system were ranked from large to small as follows: the water curtain borehole pressures, length, spacing and dip angle. The traditional numerical model relies on the assumption of homogeneous hydraulic properties of the rock mass surrounding the storage caverns. While the influence of the spatial variability of the hydraulic conductivity on the water-sealed performance of the water curtain system in underground oil storage has been ignored.

To evaluate the performance of the water curtain system accurately, the spatial variability of hydraulic conductivity must be carefully simulated. Classical widely-used probabilistic approaches treat the geotechnical parameter of concern as a single random variable, ignoring its spatial correlation^12^. To more accurately reflect the geotechnical properties, Vanmarcke et al.^13^ introduced the random field theory into the geotechnical profession to study the spatial variability of geotechnical parameters. The random field is essentially a random (or stochastic) process consisting of indexed (i.e. ordered according to one or more reference directions) random variables^14^. At present, the random field theory has been widely used in geotechnical engineering, such as slopes^15^, dam^16–18^, and underground space engineering^19–21^. Because the quantity of available measurements is usually not large enough, especially in the horizontal direction, the input data used to characterise the material properties in these studies was hypothetical. In contrast, limited research exists related to the optimisation design of water curtain system in underground oil storage based on reliability analysis methods.

At present, reliability analysis methods commonly used in geotechnical engineering are the first-order reliability method (FORM), second-order reliability method (SORM), Monte Carlo simulations (MCS), and the point estimate method^22,23^. Because of its simplicity, high accuracy, and adaptability to arbitrary distributions and nonlinear performance functions, MCS has been widely used for reliability analysis in geotechnical engineering^24,25^.

According to the mentioned studies, considering the spatial variability of hydraulic conductivity, the key challenging issue is how to integrate numerical simulation methods and field test results to evaluate water curtain performance in underground oil storage. This study aims to establish a probabilistic analysis methodology for water curtain performance in underground oil storage considering the spatial variability of the hydraulic conductivity of the surrounding rock based on field data. The spatial variability of the surrounding rock properties was studied using spatial statistical analysis of the available dataset obtained from the field test (e.g., borehole water injection test and water curtain boreholes injection fall-off test). Anisotropic random fields, representing the spatial variability of hydraulic conductivity, were established through spatial statistical analysis and introduced into the finite element model of underground oil storage for water-sealed reliability analysis. The water-sealed performance of different water curtain system schemes (different water curtain borehole spacings, pressures, and distances from the cavern) was studied using MCS. Finally, some recommendations were provided for the optimal design of the water curtain system. This study provides valuable insights and a theoretical basis for the optimisation of water curtain system design parameters for underground water-sealed oil storage in the face of the spatial variability of hydraulic conductivity.

## Methods

**Figure 1:**
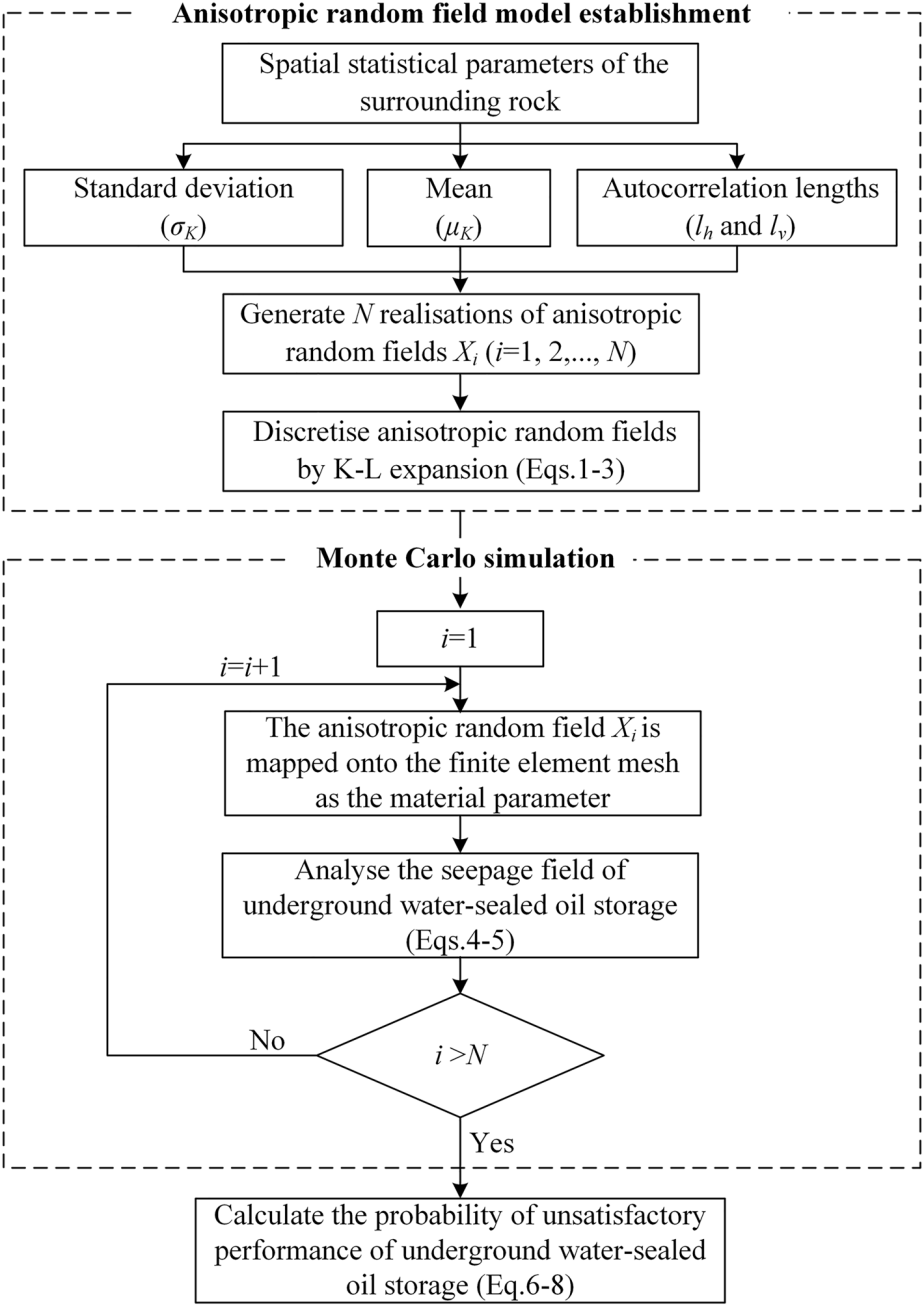
Flowchart of water-sealed reliability analysis of underground oil storage.

In this study, by coupling the random field theory with the finite element method, within a Monte Carlo framework, the water-sealed reliability of underground oil storage was analysed considering the spatial variability of hydraulic conductivity. From an engineering point of view, the uncertainty quantification of geotechnical parameters can not only estimate the risk, but also make optimal decisions within an uncertain framework. Based on the spatial statistical parameters of hydraulic conductivity, *N* realisations of anisotropic random fields were generated. The anisotropic random fields were discretised via the Karhunen–Loeve (K–L) expansion and introduced into the finite element model of underground water-sealed oil storage for seepage analysis. Based on the performance function of the cavern tightness, the probability of unsatisfactory performance was calculated using the MCS for *N* realisations. A flowchart is shown in Fig. 1.

### Random field theory

Because it is practically difficult to precisely characterise the spatial distribution of geotechnical parameters at the site, random fields are used to concisely express the spatial variability of the geotechnical properties. A random field model is completely described by its mean, variance, and autocorrelation function. In this study, an exponential two-dimensional autocorrelation function was adopted with different autocorrelation lengths in the horizontal and vertical directions to describe the anisotropic heterogeneity of hydraulic conductivity^26^:

1 $$ \rho (\tau_{1} ,\tau_{2} ) = \exp ( - \left[ {\left( {\frac{{\left| {\tau_{1} } \right|}}{{l_{h} }}} \right) + \left( {\frac{{\left| {\tau_{2} } \right|}}{{l_{v} }}} \right)} \right] $$

where *τ*_1_ and *τ*_2_ are the lag distances in the horizontal and vertical coordinate directions, respectively, and *l*_*h*_ and *l*_*v*_ are the horizontal and vertical autocorrelation lengths, respectively.

Because of the discrete nature of finite element methods, a continuous-parameter random field must also be discretised into random variables. This process is commonly known as the discretisation of a random field. K–L expansion has been widely used in geotechnical engineering because it requires the fewest random variables for a prescribed level of accuracy^27,28^. In this study, the random field was discretised via K–L expansion. The series expansion of the random field *H*(*x*, *y*, *θ*) is expressed as follows:

2 $$ H(x,y,\theta ) = \mu { + }\sigma \sum\limits_{i = 1}^{\infty } {\sqrt {\lambda_{i} } f_{i} (x,y)} \xi_{i} (\theta ) \cong \mu { + }\sigma \sum\limits_{i = 1}^{M} {\sqrt {\lambda_{i} } f_{i} (x,y)} \xi_{i} (\theta ) $$

where *x* and *y* are the coordinates of any point in the random field; *µ* and *σ* are the mean value and standard deviation, respectively, *ξ*_*i*_ (*θ*) is a set of orthogonal random coefficients, and *M* is the number of K–L expansion terms to be retained. *λ*_*i*_ and *f*_*i*_(*x*, *y*) are the eigenvalues and eigenfunctions of the two-dimensional autocorrelation function *ρ*(*τ*_1*,*_* τ*_2_), respectively.

It should be noted that the desired accuracy of the simulated random field is determined by the number of truncated terms *M* in Eq. (2), which relies on the ratio of the correlation distance to the geometric size. Several studies^29,30^ took the ratio of the expected energy, *ε*, as a measure of the accuracy of the truncated series, which is defined as

3 $$ \begin{aligned} \varepsilon & = \int\limits_{\Omega } {E\left[ {\upsigma \sum\limits_{i = 1}^{M} {\sqrt \lambda f_{i} (x,y)\xi_{i} (\theta )} } \right]}^{2} dxdy/E\left[ {\upsigma \sum\limits_{i = 1}^{\infty } {\sqrt {\lambda_{i} } f_{i} (x,y)\xi_{i} (\theta )} } \right]^{2} dxdy \\ & = \sum\limits_{i = 1}^{M} {\lambda_{i} /\sum\limits_{i = 1}^{\infty } {\lambda_{i} } } \\ \end{aligned} $$

where the eigenvalues *λ*_*i*_ are sorted in a descending order. The ratio of the expected energy *ε* exceeds a threshold value (e.g., 0.95) at approximately *M* = 3000, and the simulated random field reaches the desired accuracy.

### Governing equation

The project site for this study was a strategic oil storage cavern. In the operating phase, the seepage field around an underground water-sealed oil storage unit can be regarded as a relative equilibrium state^31^. Thus, a steady-state model approximately reflects the long-term water-sealed reliability of underground oil storage. The underground seepage field is subject to the seepage continuity equation and Darcy’s law:

4 $$ \nabla \cdot (\rho u) = Q_{m} $$

5 $$ u = - \frac{k}{\mu }(\nabla p + \rho g\nabla D) $$

where *Q*_*m*_ is the mass source term [kg/(m^3^ s)], *u* is the Darcy velocity or specific discharge vector (m/s), *k* is the permeability of the porous medium (m^2^), *μ* is the fluid dynamic viscosity (Pa s), *p* is the fluid pressure (Pa), *ρ* is the density (kg/m^3^), and $\nabla D$ is a unit vector in the direction of gravity.

### Water-sealed reliability analysis

Previous studies have shown that a water curtain system ensures the groundwater pressure around the storage caverns is greater than the oil pressure in the caverns during the operating phase, which maintains water-sealed reliability^32^. The performance function of the cavern tightness is expressed as follows:

6 $$ G(n,X_{i} ) = \frac{{P(n,X_{i} )}}{{P_{g} (n)}} - 1 $$

where *X*_*i*_ (i = 1, 2, …, *N*) represents a random vector of the input variables, which corresponds to the random variables used to discretise the random fields using the K–L expansion in Eq. (2); *N* is the number of realisations of the random field; and *n* is the monitoring point location index. *P*(*n*, *X*_*i*_) (*n* = 1, 2…, 33) is the pore water pressure at monitoring point *n* around the caverns when the input variable is *X*_*i*_ (MPa) and *P*_*g*_(*n*) is the stable oil (gas) pressure at the monitoring point *n* in the caverns (MPa).

Based on the performance function of cavern tightness, the probability of unsatisfactory performance was calculated using MCS. The initial diffusion of crude oil is from the cavern boundary, and the vicinity of caverns are our concerned area. When the value of *G* (*n*, *X*_*i*_) at the monitoring point *n* is lower than a predefined threshold, the oil–water interface at the monitoring point *n* moves outward, but it is not sufficiently low to indicate catastrophic crude oil leakage. To distinguish between catastrophic failure and less significant performance problems, the probability of unsatisfactory performance was used to evaluate the water-sealed reliability of the underground oil storage^33^. The probability of unsatisfactory performance is often expressed as:

7 $$ p_{f} (n) = p\left[ {G(n,X_{i} ) < 0} \right] = \frac{1}{N}\sum\limits_{i = 1}^{N} {I\left[ {G(n,X_{i} ) < 0} \right]} $$

8 $$ I\left[ \cdot \right] = \left\{ {\begin{array}{*{20}c} {1,\;\;\;G(n,X_{i} ) < 0} \\ {0,\;\;\;G(n,X_{i} ) \ge 0} \\ \end{array} } \right. $$

where *I*[*·*] is the indicator function used to describe whether the water-sealed performance of underground oil storage is satisfactory.

## Spatial variability of seepage characteristics

### Hydraulic conductivity distribution

**Figure 2:**
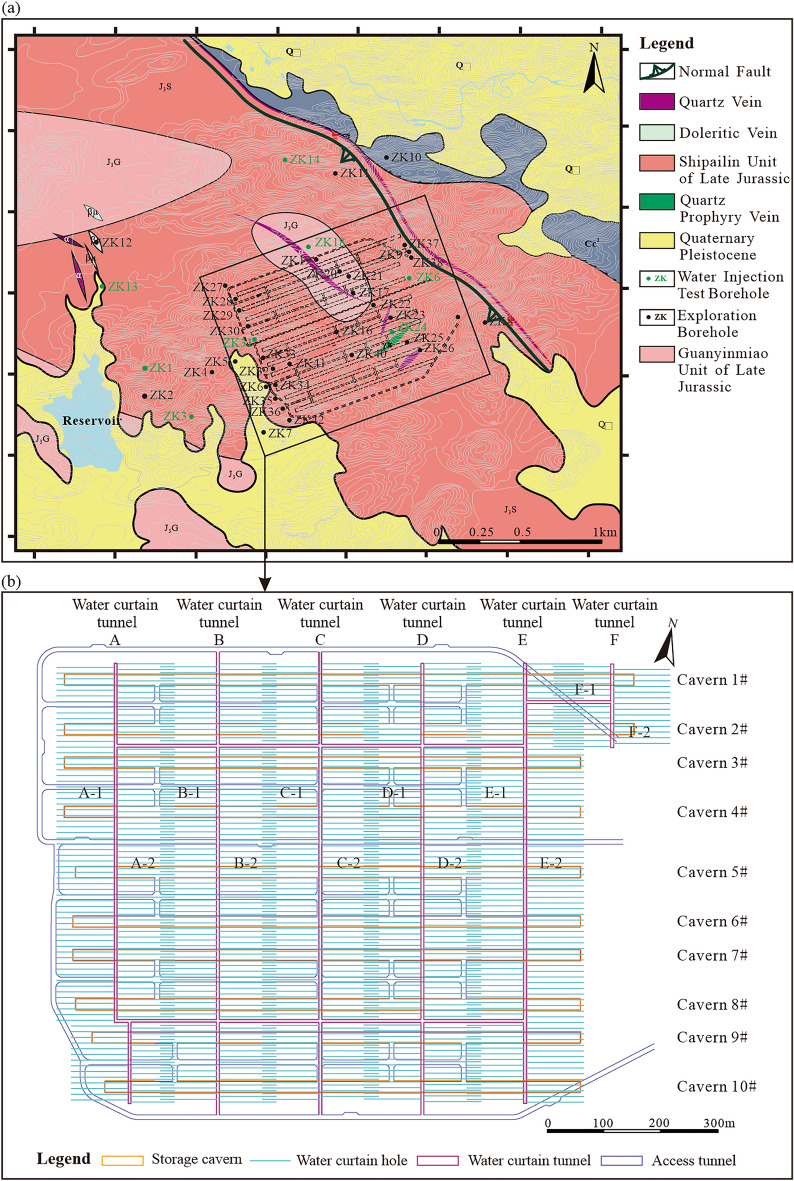
Geological condition of the study area and layout of the underground water-sealed oil storage cavern: (**a**) geological structure map and location of boreholes (adapted from^34^ and (**b**) sketch map of the study project.

**Figure 3:**
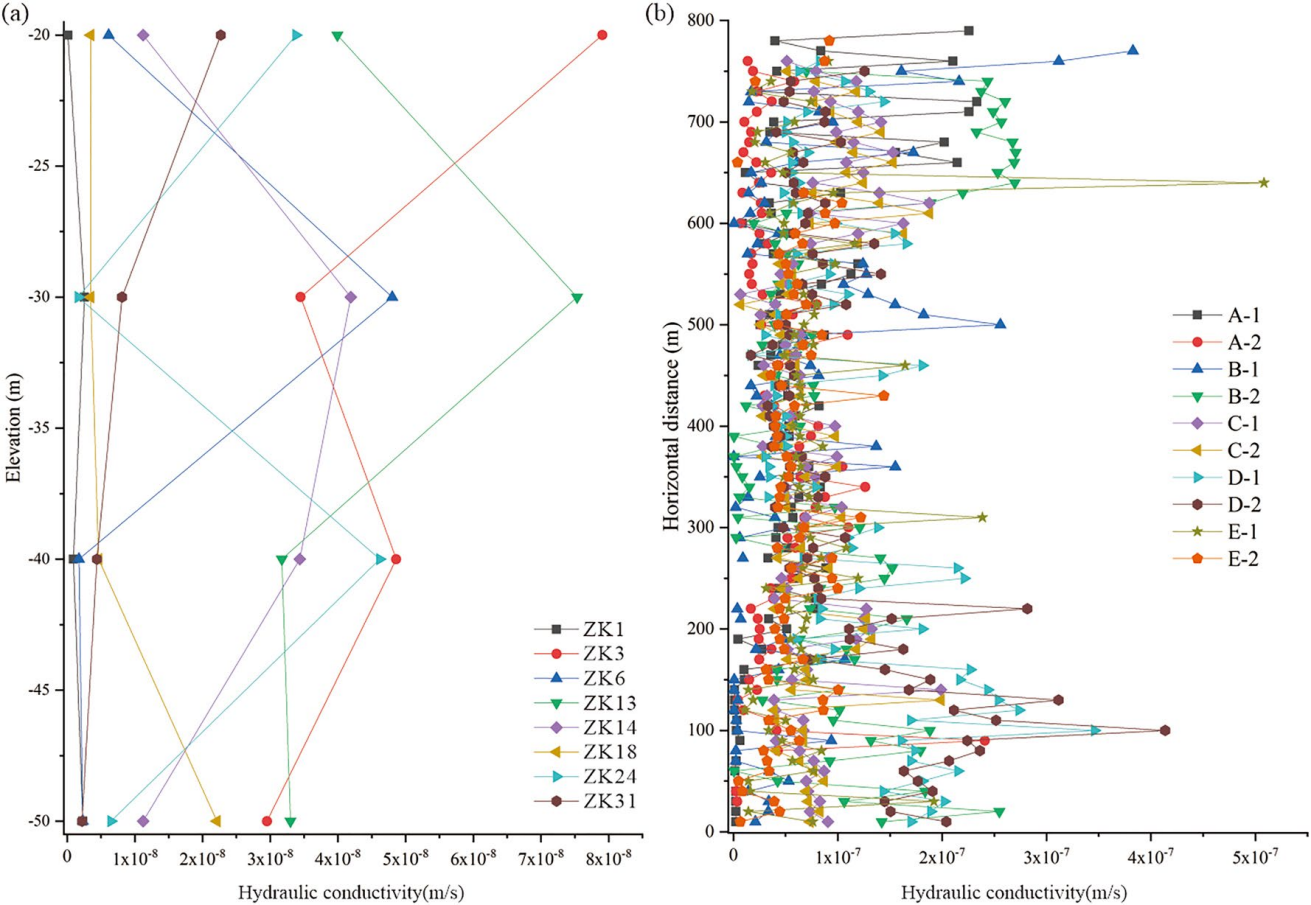
Distribution of hydraulic conductivity at the site: (**a**) vertical distribution of hydraulic conductivity at the site and (**b**) horizontal distribution of hydraulic conductivity at the site.

The underground water-sealed oil storage in this study was located on the southeast coast of China. The strata of the study area included granite rocks of the Carboniferous Ceshui Formation (Cc1) and the Quaternary Pleistocene (Qω) (Fig. 2). To explore the seepage characteristics of the surrounding rock at the site, a large number of field tests were conducted to determine the hydraulic conductivity of the surrounding rock. Two main datasets were available in this study: borehole water injection tests and water curtain borehole injection fall-off test. Before the construction of the storage caverns, water injection tests were conducted at eight boreholes to measure the hydraulic conductivity along with the depth at the site (Figs. 2a, 3a). After the construction of the water curtain system was completed, injection fall-off tests were conducted at 784 water curtain boreholes to measure the hydraulic conductivity along with the horizontal direction at the site (Figs. 2b, 3b). These datasets provided a strong basis for exploring the spatial variability of seepage characteristics of the surrounding rock at the site.

**Figure 4:**
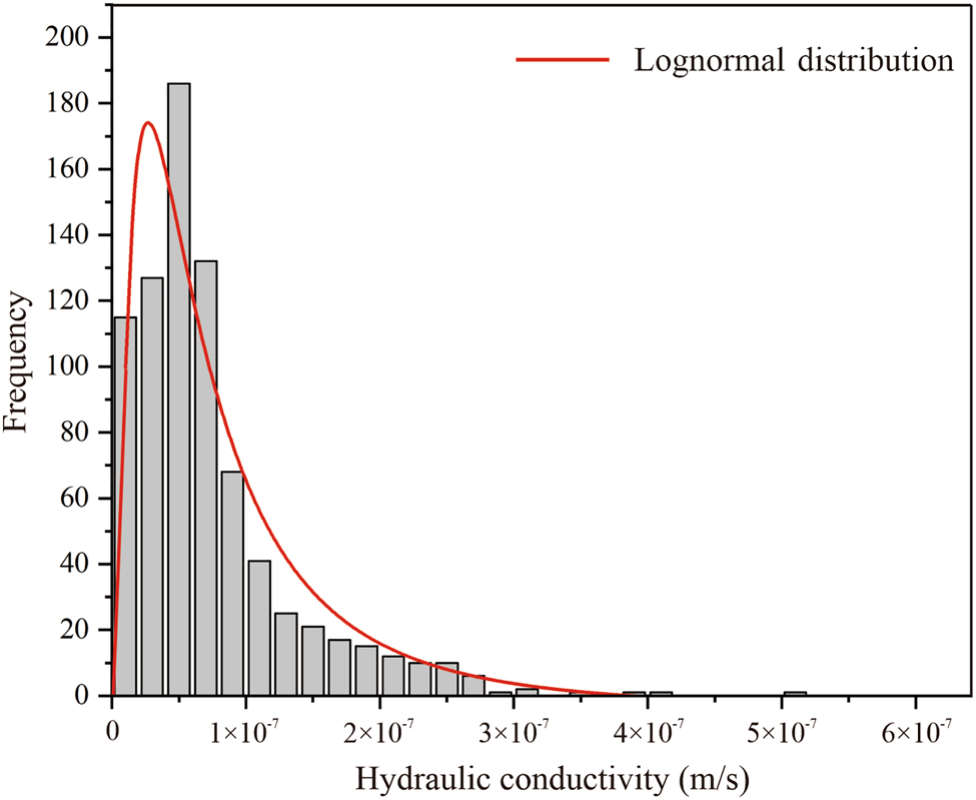
Frequency histogram of hydraulic conductivity.

The 792 hydraulic conductivity samples extracted from the boreholes were used to produce a frequency histogram, as shown in Fig. 4. It was observed that the hydraulic conductivity at the site approximately obeyed a lognormal distribution. Moreover, the hydraulic conductivity is always nonnegative. Thus, for random field modelling, it was appropriate to assume that the hydraulic conductivity follows a lognormal distribution^35^. The calculated mean (*μ*_ln*K*_) and variance (*σ*^2^_ln*K*_) of the logarithm of the samples (ln*K*) were − 16.87 and 1.31, respectively. The mean (*μ*_*K*_), variance (*σ*^2^_*K*_), and coefficient of variation (*δ*_*K*_) of the lognormal distribution of the hydraulic conductivity are expressed as follows^36^:

9 $$ \mu_{K} = \exp (\mu_{\ln K} + \sigma_{\ln K}^{2} /2) $$

10 $$ \sigma_{K}^{2} = \left[ {\exp (\sigma_{\ln K}^{2} ) - 1} \right]\exp (2\mu_{\ln K} + \sigma_{\ln K}^{2} ) $$

11 $$ \delta_{K} = \frac{{\sigma_{K} }}{{\mu_{K} }} $$

The calculated mean, standard deviation, and coefficient of variation of hydraulic conductivity were 9.04 × 10^−8^ m/s, 1.48 × 10^−7^ m/s, and 165%, respectively. According to previous studies^37^, the hydraulic conductivity of fractured granite is generally in the range of 8 × 10^−9^–3 × 10^−4^ m/s, and the coefficient of variation is large. The results of this study were within the normal range.

### Spatial correlation of hydraulic conductivity

**Figure 5:**
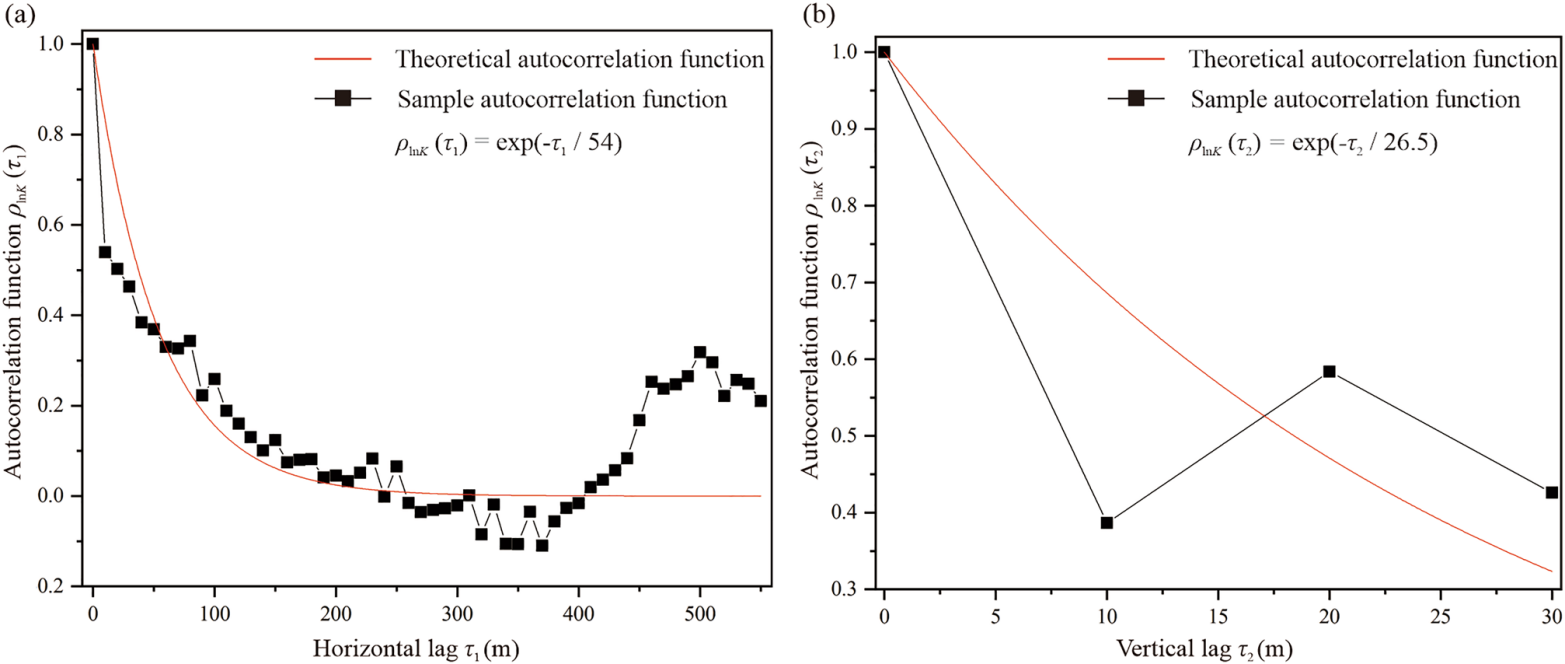
Fitting results for autocorrelation function of ln*K*: (**a**) horizontal autocorrelation function and (**b**) vertical autocorrelation function.

The autocorrelation length, defined as the distance at which the autocorrelation function decays to 1/e (e is the base of natural logarithms), is used to describe the spatial extent within which the rock properties show a strong correlation^38^. In this study, based on a large number of field test samples, the spatial correlation of the hydraulic conductivity of surrounding rock was explored. Based on the samples from eight boreholes along with the depth and 784 water curtain boreholes along with the horizontal direction, the autocorrelation lengths of the random field were calculated using the correlation function method^39^. Using the least squares method, the autocorrelation function of ln*K* was fitted with the theoretical autocorrelation function (Fig. 5).

The results showed that the horizontal autocorrelation length (*l*_*h*_) and vertical autocorrelation length (*l*_*v*_) of ln*K* were 54 m and 26.5 m, respectively. The calculated autocorrelation lengths have physical meaning and reflect the spatial correlation of the hydraulic conductivity in the depth and horizontal direction of the site. It was observed that the horizontal autocorrelation length was greater than the vertical autocorrelation length. The vertical autocorrelation function of the hydraulic conductivity fluctuated considerably owing to the lack of a spatially dense sample dataset in the vertical direction. Therefore, the following analysis investigates the effects of the autocorrelation length of hydraulic conductivity on the water-sealed performance in underground oil storage.

## Water curtain performance assessment considering probabilistic mechanism

### Stochastic model

**Figure 6:**
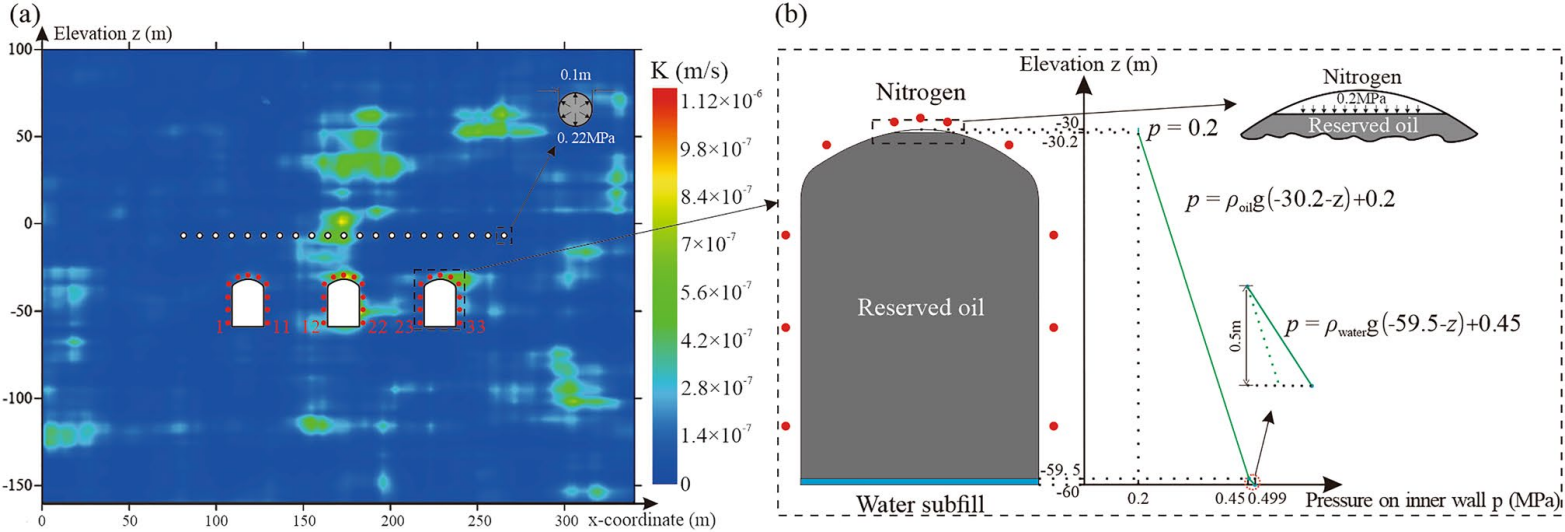
Numerical model of underground water-sealed oil storage for one realisation of the anisotropic random field: (**a**) two-dimensional vertical section model and (**b**) boundary of the oil storage cavern. The figure was generated using the software COMSOL Multiphysics, version 5.3 (https://www.comsol.com/).

For simplicity, and to avoid the numerical computational burden, a two-dimensional vertical section finite element model of three oil storage caverns was established to simulate the underground seepage field (Fig. 6). According to the spatial statistical analysis of the field test data, two-dimensional anisotropic random fields representing hydraulic conductivity were established and introduced into the numerical model for seepage analysis. The scale of the numerical model was 340 m (length) and 260 m (height). The model contained 15,828 elements. The depth of the oil storage caverns from the land surface was 130 m. The caverns had a width of 20 m, height of 30 m, and spacing of 40 m. A total of 33 monitoring points were arranged around the three caverns (Fig. 6a). The lateral boundaries were prescribed with the mean underground water level observed in the site investigations (e.g. 100 m). The lower boundaries were considered impermeable. The boundary of the oil storage cavern satisfied the boundary of the fixed water level. A fixed water bed with a thickness of 0.5 m was installed below the cavern, and a 0.2 MPa nitrogen atmosphere was maintained above the cavern (Fig. 6b).

We assumed that other hydraulic parameters spatial variability has minimal effects on water-sealed reliability of underground oil storage in this study, and they were set to be uniform and determined. Based on previous studies and field investigations^40^, the dynamic viscosity (*μ*) was 1.005 × 10^−3^ Pa s [Eq. (5)]. The densities of water and oil were 1000 kg/m^3^ and 878 kg/m^3^, respectively. To investigate the effects of different water curtain holes parameters on the water-sealed reliability of underground oil storage, the water curtain system was simplified into a series of water curtain holes, and 25 numerical cases were developed and analysed. Three variable controlling parameters were studied. Here are three simulation scenarios: (a) different spacings of water curtain boreholes: 10 m, 15 m, 20 m, 25 m, 30 m, 35 m, 40 m, 45 m, and 50 m; (b) different distances between water curtain boreholes and caverns: 10 m, 15 m, 20 m, 26.5 m, 30 m, 35 m, and 40 m; and (c) different pressures of the water curtain holes: 0.1 MPa, 0.15 MPa, 0.2 MPa, 0.22 MPa, 0.3 MPa, 0.35 MPa, 0.4 MPa, 0.45 MPa, and 0.5 MPa. In addition, in scenario d, another six additional numerical cases with different vertical autocorrelation lengths (*l*_*v *_= 6.5 m, 16.5 m, 26.5 m, 36.5 m, 46.5 m, 54 m) were adopted to explore the effect of the spatial correlation of hydraulic conductivity on the water-sealed reliability of underground oil storage. For all other cases, a pressure of 0.22 MPa (scenarios a, b, and d), a spacing of 10 m (scenarios b, c, and d), and a distance of 26.5 m from the caverns (scenarios a, c, and d) were maintained.

### Water-sealed reliability analysis

#### Seepage analysis

**Figure 7:**
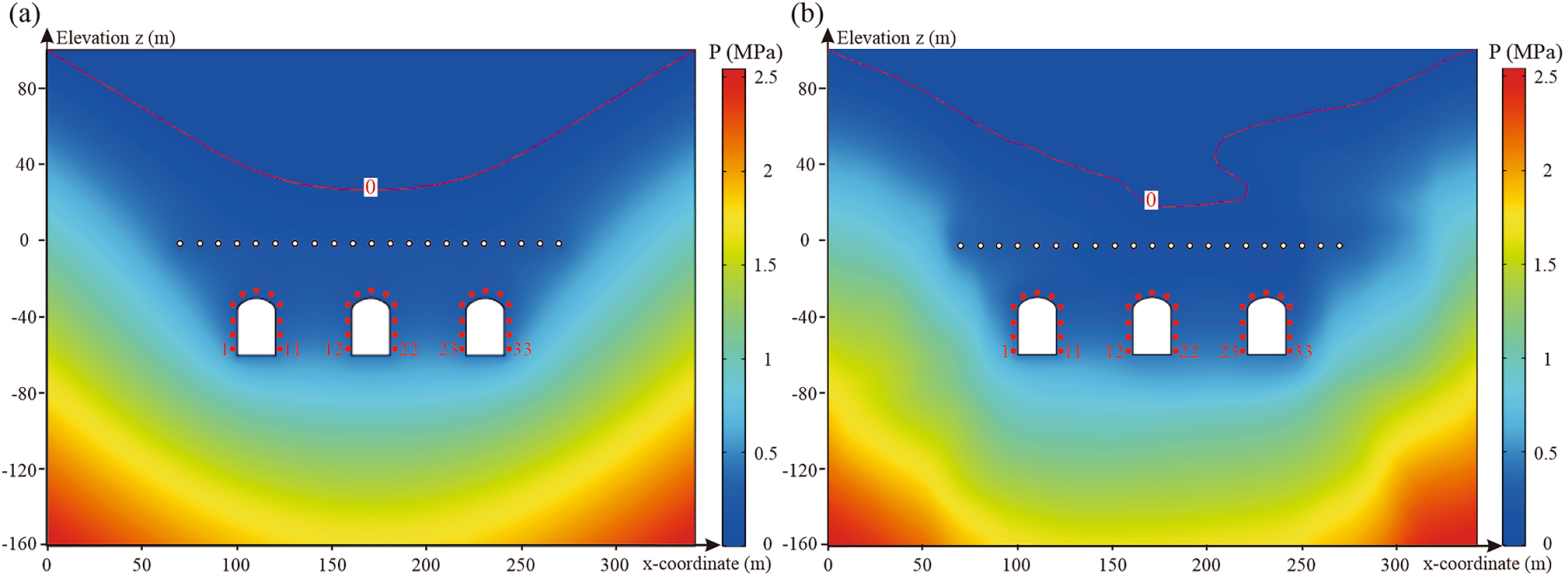
Pore water pressure distribution of underground water-sealed oil storage: (**a**) homogeneous hydraulic conductivity and (**b**) heterogeneous hydraulic conductivity with anisotropic correlation. The figure was generated using the software COMSOL Multiphysics, version 5.3 (https://www.comsol.com/).

**Figure 8:**
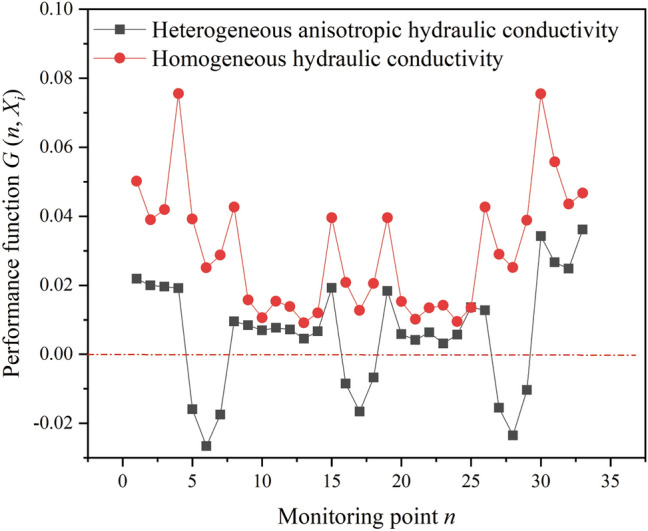
Water-sealed reliability of monitoring points around the caverns.

A seepage analysis of underground water-sealed oil storage in homogeneous and heterogeneous media was conducted to investigate the influence of the spatial variability of hydraulic conductivity on the water-sealed reliability of underground oil storage. In homogeneous media, the hydraulic conductivity is uniform, and its value is represented by the mean of 792 hydraulic conductivity samples (i.e. *K* = 9.04 × 10^−8^ m/s). In heterogeneous media, hydraulic conductivity varies spatially. Based on the spatial statistical parameters of the hydraulic conductivity obtained in “Spatial variability of seepage characteristics” section  (*μ*_*K*_ = 9.04 × 10^−8^ m/s, *σ*_*K*_ = 1.48 × 10^−7^ m/s, *l*_*h*_ = 54 m, and *l*_*v *_= 26.5 m), one realisation of the anisotropic random field representing the spatial variability of hydraulic conductivity, was generated and introduced into the finite element model of underground oil storage for seepage analysis. The pore water pressure distribution of the underground water-sealed oil storage and the value of the performance function (*G* (*n*, *X*_*i*_)) at the monitoring points around the caverns are shown in Figs. 7 and 8.

As shown in Fig. 7, the spatial variability of the hydraulic conductivity had a significant effect on the pore water pressure distribution in the seepage field. In heterogeneous media, the pore water pressure distribution was not asymmetric. The groundwater level above the caverns and the pore water pressure around the caverns fluctuated considerably. According to Fig. 8, the values of *G* (*n*, *X*_*i*_) at the monitoring points around the caverns are all greater than zero in homogeneous media. However, in heterogeneous media, all *G* (*n*, *X*_*i*_) values at the monitoring points were reduced. Thus, ignoring the spatial variability of the hydraulic conductivity may lead to an overestimation of the water-sealed reliability of the underground oil storage unit.

**Figure 9:**
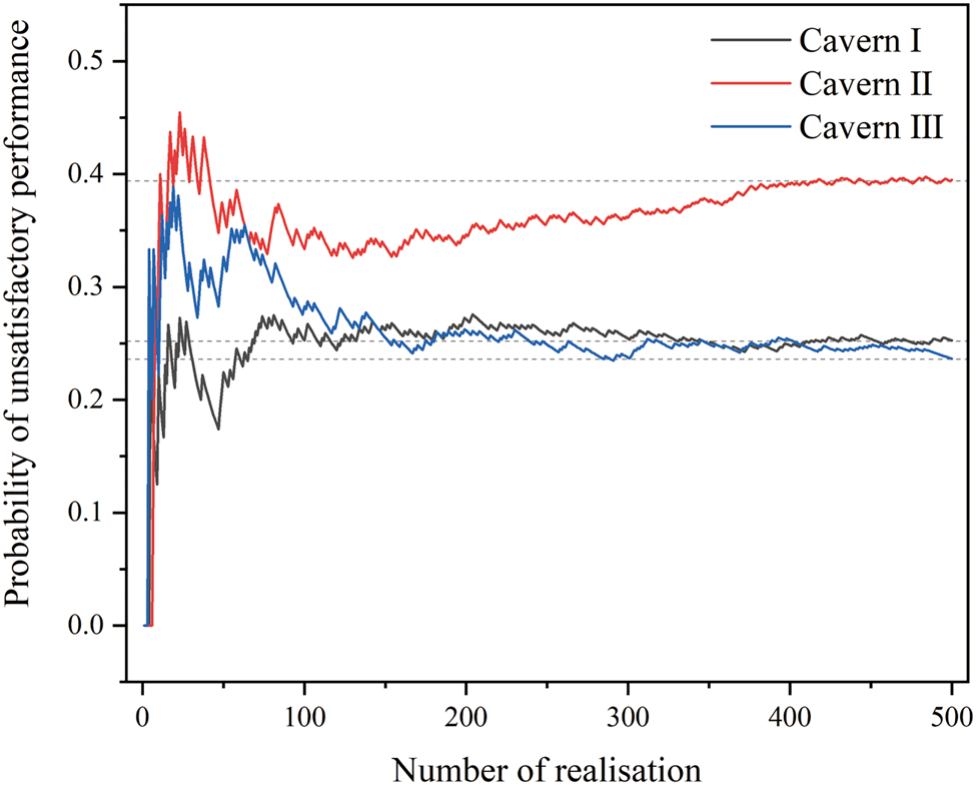
Effect of number of realisations on probability of unsatisfactory performance.

The values of *G* (*n*, *X*_*i*_) at the monitoring points above the caverns were less than zero, and the values of *G* (*n*, *X*_*i*_) at the monitoring points at the top of the three caverns were the smallest. In addition, owing to the interaction between the caverns, the spatial variability of the hydraulic conductivity has little effect on the *G* (*n*, *X*_*i*_) values at the monitoring points between the caverns. It can be concluded that the regions at the top of the three caverns are the likeliest to have unsatisfactory water-sealed performance. Accordingly, the water-sealed reliability at the monitoring points at the top of the caverns was investigated. When the number of realisations of the anisotropic random field approached 500 in the MCS, the calculated probability of unsatisfactory performance of the three caverns became stable (Fig. 9). That is, MCS had a sufficient number of samples.

#### Effect of the autocorrelation length

**Figure 10:**
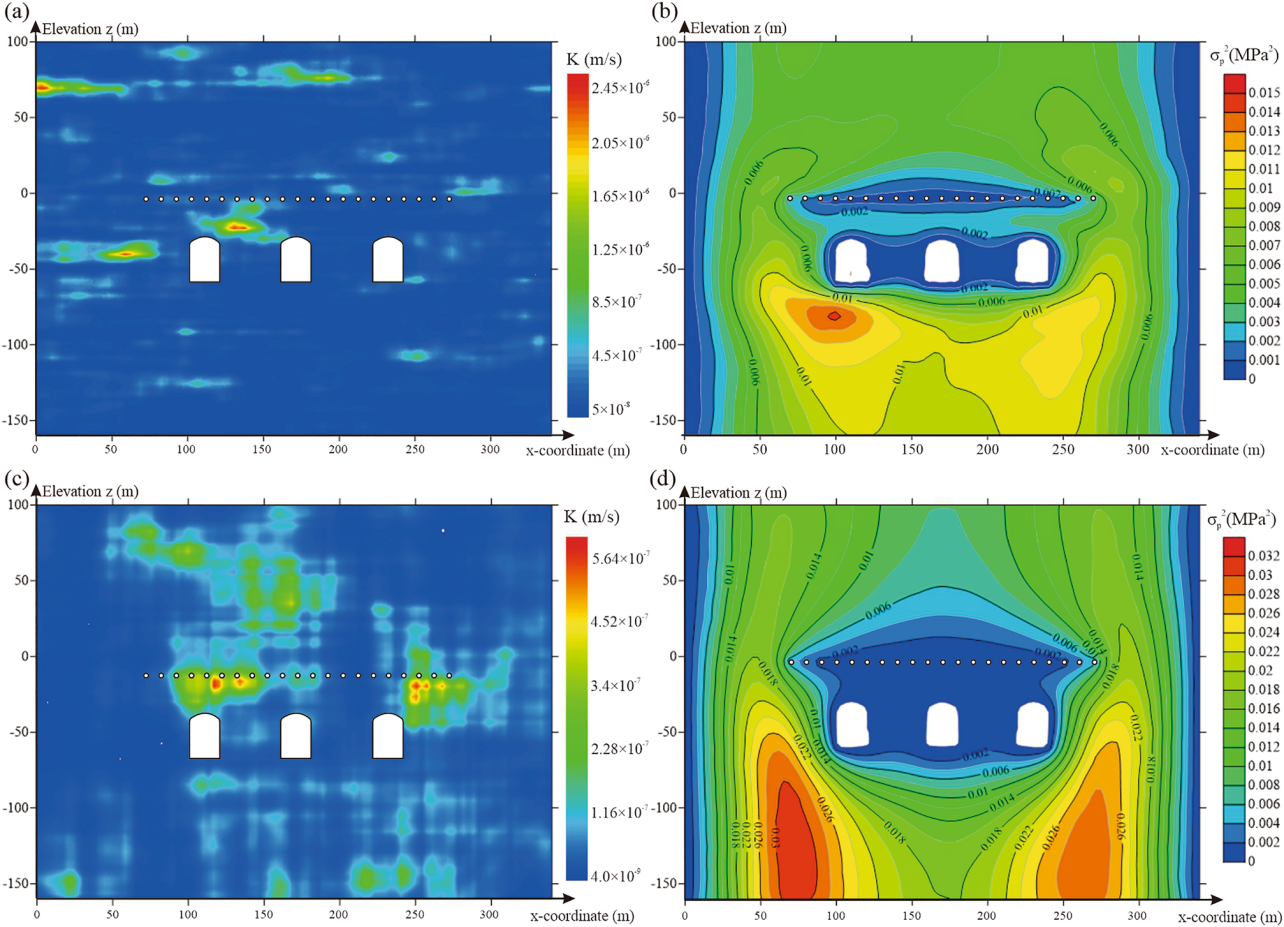
Random field and pore water pressure distribution with different vertical autocorrelation lengths: (**a**) one realisation of the random field with a vertical autocorrelation length of 6.5 m, (**b**) variance distribution of pore water pressure for 500 realisations of the random field with a vertical autocorrelation length of 6.5 m, (**c**) one realisation of the random field with a vertical autocorrelation length of 54 m and (**d**) variance distribution of pore water pressure for 500 realisations of the random field with a vertical autocorrelation length of 54 m. The figure was generated using the software COMSOL Multiphysics, version 5.3 (https://www.comsol.com/).

The autocorrelation lengths are important for describing the anisotropy of the heterogeneity of geotechnical parameters. Thus, the influence of the autocorrelation length on the water-sealed reliability of underground oil storage was studied. Based on the spatial statistical parameters of the hydraulic conductivity obtained in “Spatial variability of seepage characteristics” section (*μ*_*K*_ = 9.04 × 10^−8^ m/s, *σ*_*K*_ = 1.48 × 10^−7^ m/s, *l*_*h*_ = 54 m), random fields were generated with different vertical autocorrelation lengths. An extreme case, with equal horizontal and vertical autocorrelation lengths, corresponded to the isotropy of the heterogeneity of hydraulic conductivity. For each value of the autocorrelation length, 500 realisations of the random fields representing hydraulic conductivity were simulated (Fig. 10a,c). As shown in Fig. 10a,c, as the vertical autocorrelation length decreases, the ratio of the horizontal autocorrelation length to the vertical autocorrelation length increases, and the spatial heterogeneity of hydraulic conductivity in the random field increases.

Random fields were introduced into the numerical model of the underground water-sealed oil storage; the variance of pore water pressure with different water curtain borehole pressures for 500 realisations of the random fields was calculated and is presented in Fig. 10b,d. As shown in Fig. 10, the vertical autocorrelation length has a significant effect on the pore water pressure distribution in the seepage field of underground oil storage. The results showed that as the vertical autocorrelation length decreases, the variance of the pore water pressure between the caverns and water curtain boreholes increased and the uncertainty of the water pressure increased.

**Figure 11:**
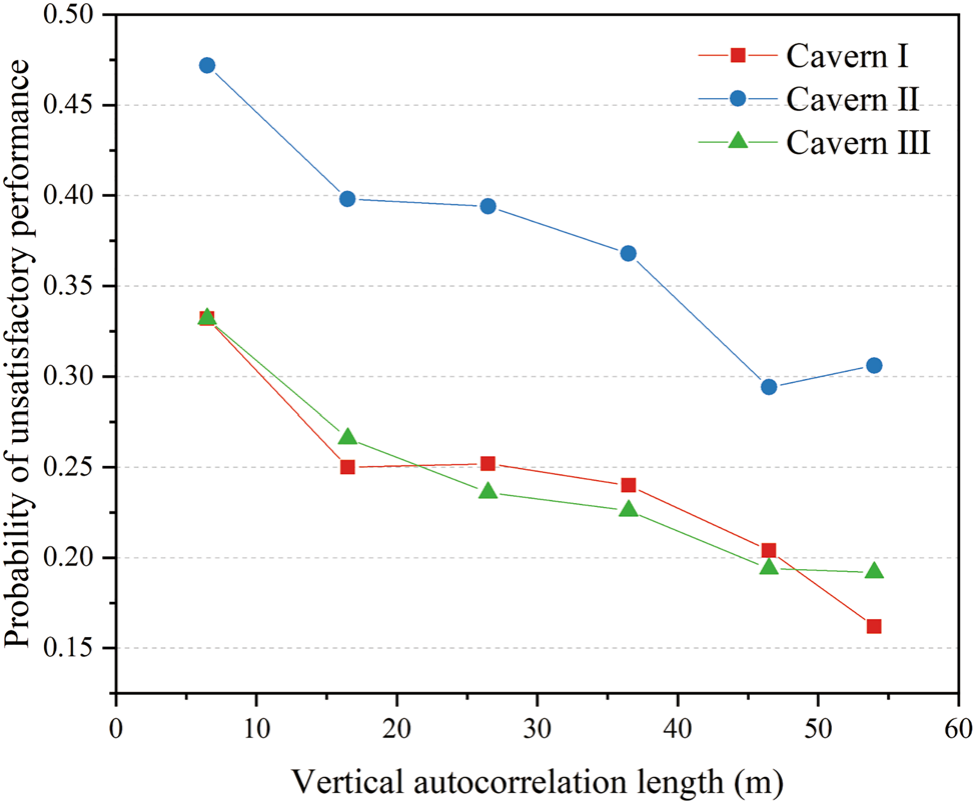
Probability of unsatisfactory performance with different vertical autocorrelation length.

The probability of unsatisfactory performance at the monitoring points at the top of the three caverns with a vertical correlation length ranging from 6.5 to 54 m was calculated using MCS. The probability curves are presented in Fig. 11, which shows that the probability of unsatisfactory performance decreases with increasing vertical autocorrelation length. From a physical perspective, a higher vertical autocorrelation length indicates that the hydraulic conductivity varies only slightly with depth. Thus, as the vertical autocorrelation length increases, it is beneficial to enhance the hydraulic connectivity between the water curtain holes and the caverns to improve the water-sealed effect of the water curtain system.

### Influence of water curtain system design parameters on water-sealed reliability

#### Spacing of water curtain boreholes

**Figure 12:**
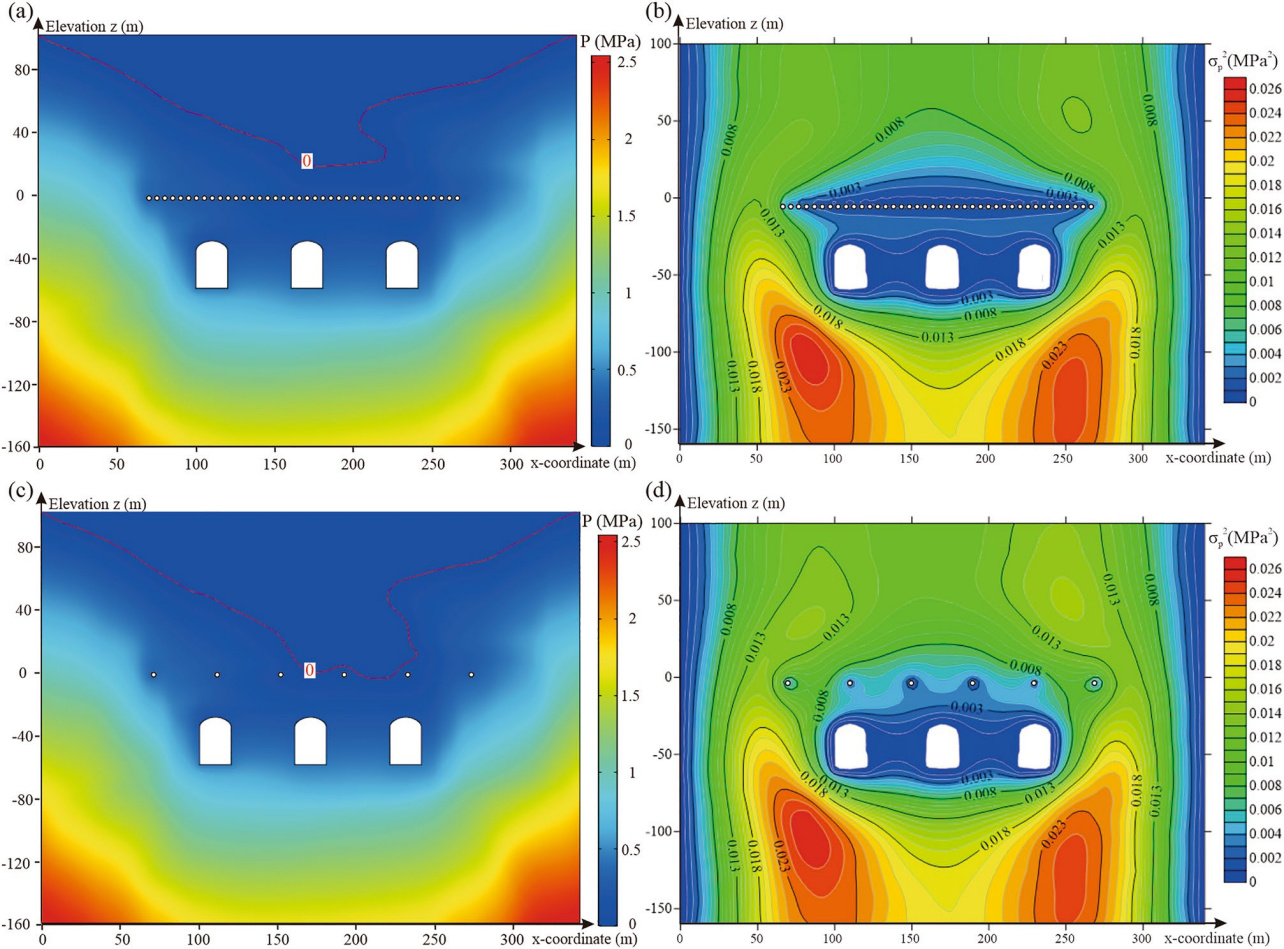
Characteristics distribution of pore water pressure with different water curtain borehole spacing: (**a**) pore water pressure distribution for one realisation of the random field with a water curtain borehole spacing of 10 m, (**b**) variance distribution of pore water pressure for 500 realisations of the random fields with a water curtain borehole spacing of 10 m, (**c**) pore water pressure distribution for one realisation of the random field with a water curtain borehole spacing of 40 m and (**d**) variance distribution of pore water pressure for 500 realisations of the random fields with a water curtain borehole spacing of 40 m. The figure was generated using the software COMSOL Multiphysics, version 5.3 (https://www.comsol.com/).

Based on the spatial statistical parameters of the hydraulic conductivity obtained in “Spatial variability of seepage characteristics” section (*μ*_*K*_ = 9.04 × 10^−8^ m/s, *σ*_*K*_ = 1.48 × 10^−7^ m/s, *l*_*h*_ = 54 m, and *l*_*v *_= 26.5 m), 500 realisations of the random fields representing hydraulic conductivity were generated. Random fields were introduced into the numerical model of the underground water-sealed oil storage with different water curtain borehole spacings for seepage analysis. Figure 12a,c show the pore water pressure distribution with different water curtain borehole spacings for one realisation of the random field. Figure 12b,d show the variance distribution of pore water pressure with different water curtain borehole spacings for 500 realisations of the random fields.

As shown in Fig. 12a,c, as the water curtain borehole spacing increases, the groundwater level above the caverns drops unevenly. When the spacing of the water curtain boreholes was 40 m, the thickness of the local water cap layer was small, which was not conducive to maintaining long-term water-sealed safety. Figure 12b,d also show that the change in the water curtain borehole spacing has a significant effect on the variance distribution of the pore water pressure above the caverns. When the spacing of the water curtain boreholes was 40 m, the variance of the pore water pressure between the water curtain holes increased significantly owing to the decrease in the number of water curtain boreholes. The pressure uncertainty between the water curtain holes increased, which impacted the water-sealed effect of the water curtain system.

**Figure 13:**
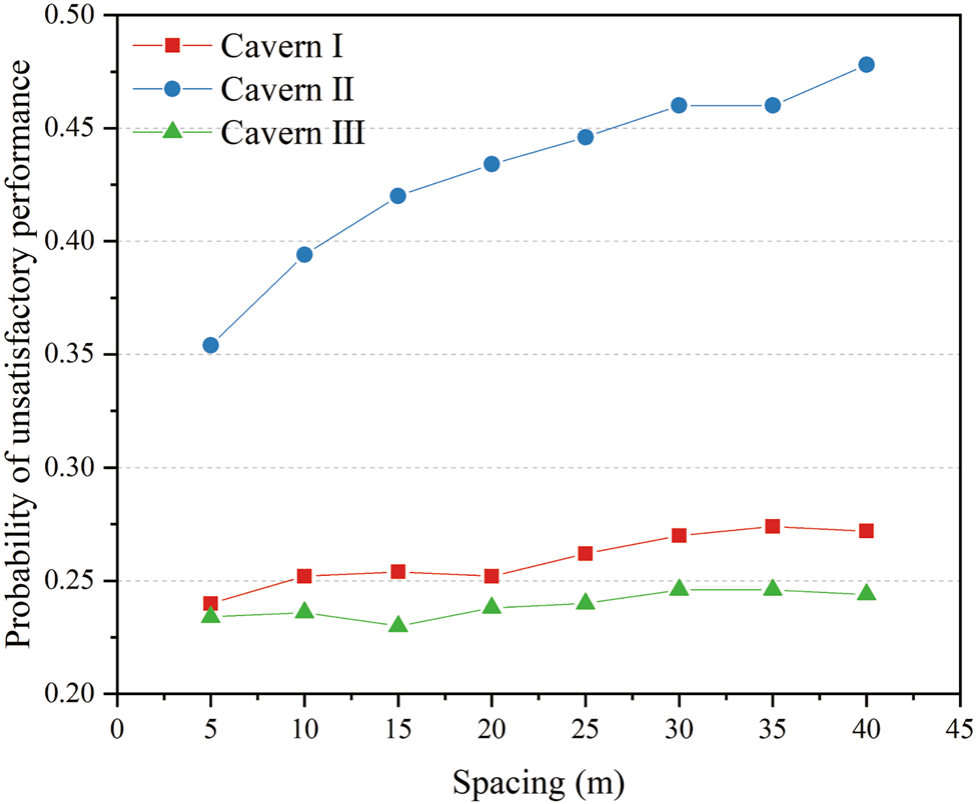
Probability of unsatisfactory performance with different water curtain borehole spacing.

For each value of the water curtain borehole spacing, the probability of unsatisfactory performance at the monitoring points at the top of the three caverns was calculated via MCS [Eqs. (6)–(8)]. Figure 13 presents the probability curves of unsatisfactory performance with a water curtain borehole spacing ranging from 5 to 40 m. As shown in Fig. 13, the water curtain borehole spacing positively correlated with the probability of unsatisfactory performance. The curves of the probability of unsatisfactory performance are concave downward. The ascending rate of the probability of unsatisfactory performance is greater with a small water curtain borehole spacing, and decreases with increased water curtain borehole spacing. With an increase in the water curtain borehole spacing, the probability of unsatisfactory performance in Cavern II changed the most. When the water curtain hole spacing increased from 5 to 40 m, the probability of unsatisfactory performance in Cavern II increased by 0.124, and the probability of unsatisfactory performance in Caverns I and III increased by 0.032 and 0.01, respectively. This indicated that the water curtain hole spacing had a greater impact on the water-sealed reliability of Cavern II. Compared with the conventional fixed interval, installing water curtain boreholes with changeable intervals may improve the utilisation efficiency of the water curtain system.

#### Distance between water curtain boreholes and caverns

**Figure 14:**
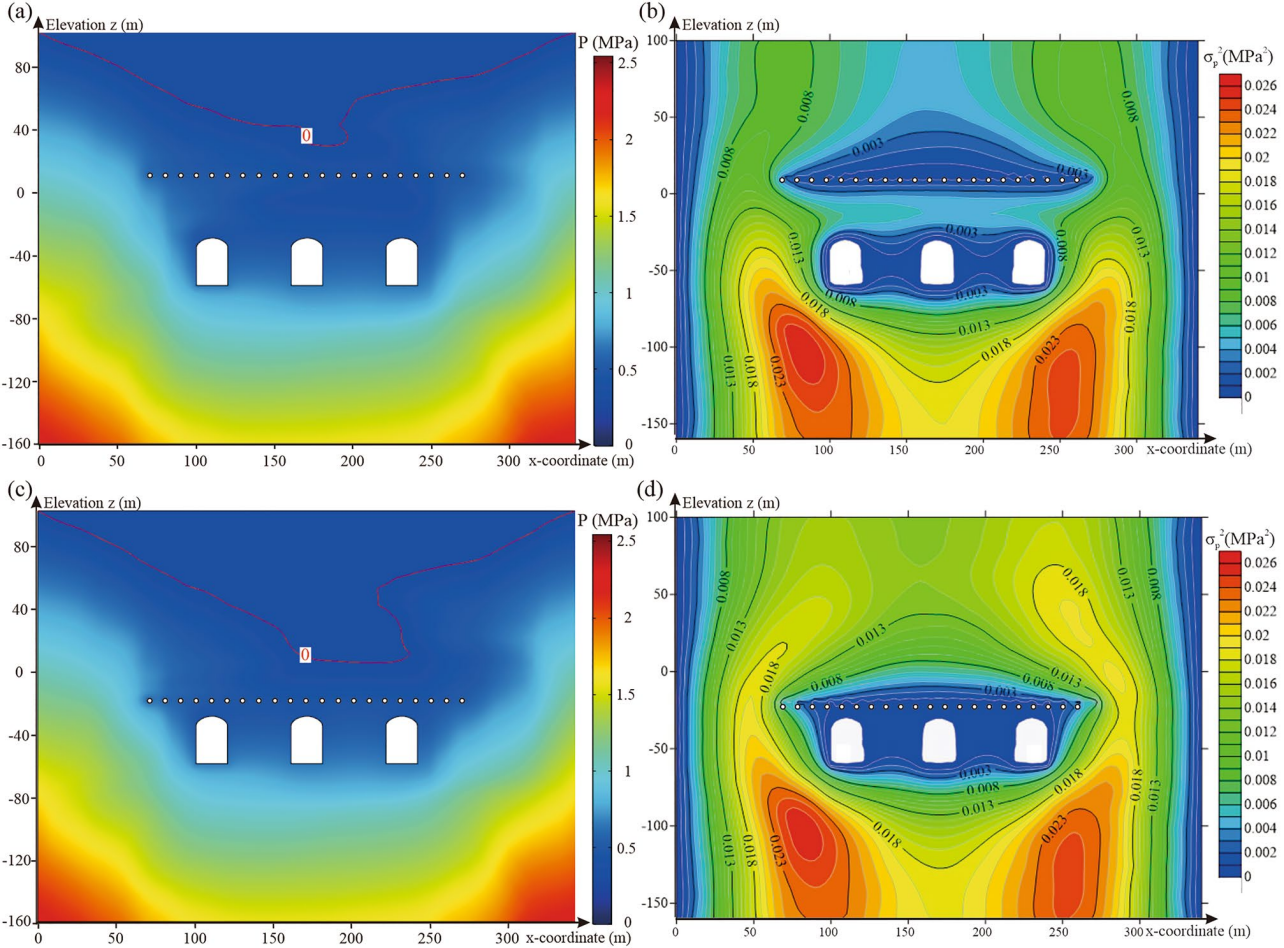
Characteristics distribution of pore water pressure for different distances between water curtain boreholes and caverns: (**a**) pore water pressure distribution for one realisation of the random field with a distance between water curtain boreholes and caverns of 40 m, (**b**) variance distribution of pore water pressure for 500 realisations of the random fields with a distance between water curtain boreholes and caverns of 40 m, (**c**) pore water pressure distribution for one realisation of the random field with a distance between water curtain boreholes and caverns of 10 m and (**d**) variance distribution of pore water pressure for 500 realisations of the random fields with a distance between water curtain boreholes and caverns of 10 m The figure was generated using the software COMSOL Multiphysics, version 5.3 (https://www.comsol.com/).

The 500 realisations of random fields were introduced into the numerical model of the underground water-sealed oil storage with different distances between the water curtain boreholes and caverns for seepage analysis. Figure 14a,c show the pore water pressure distribution with different distances between the water curtain boreholes and caverns for one realisation of the random field. The change in the distance between the water curtain boreholes and caverns considerably affects the water pressure distribution above the water curtain boreholes. As the distance between the water curtain boreholes and caverns decreased, the groundwater level dropped, but still maintained a certain distance from the water curtain holes. Figure 14b,d show the variance contour of the pore water pressure with different distances between the water curtain boreholes and caverns for 500 realisations of the random fields. The results showed that as the distance between the water curtain boreholes and caverns decreased, the variance of the pore water pressure above the water curtain boreholes and the uncertainty of the water pressure increased.

**Figure 15:**
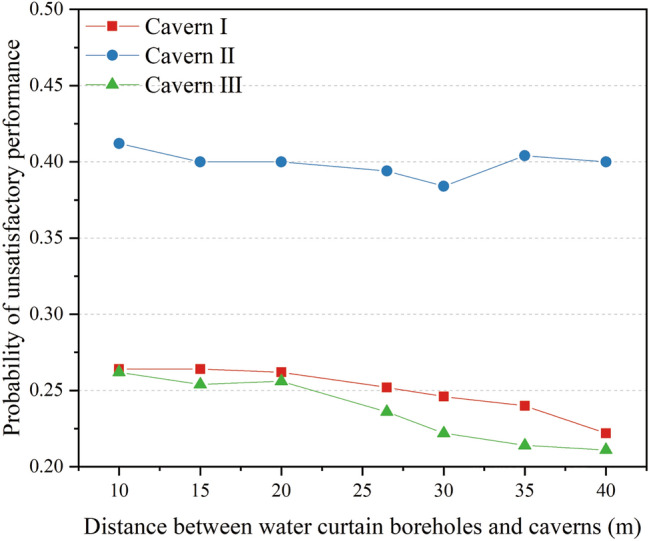
Probability of unsatisfactory performance for different distances between water curtain boreholes and caverns.

For each distance between the water curtain boreholes and caverns, the probability of unsatisfactory performance at the monitoring points at the top of the three caverns was calculated via MCS [Eqs. (6)–(8)]. Figure 15 shows the probability curves of unsatisfactory performance for distances between the water curtain boreholes and caverns ranging from 10 to 40 m. It was concluded that the distance between the water curtain boreholes and caverns negatively correlated with the probability of unsatisfactory performance. As the distance between the water curtain boreholes and caverns increased, the groundwater level raised, and the water-sealed effect of the water curtain system increased. When the distance between the water curtain boreholes and caverns increased from 10 to 40 m, the probability of unsatisfactory performance for the three caverns decreased by 0.043, 0.012, and 0.051, respectively. The results show that the distance between the water curtain holes and the caverns has the less significant affecting the water-sealed reliability of the storage cavern.

#### Pressure of water curtain boreholes

**Figure 16:**
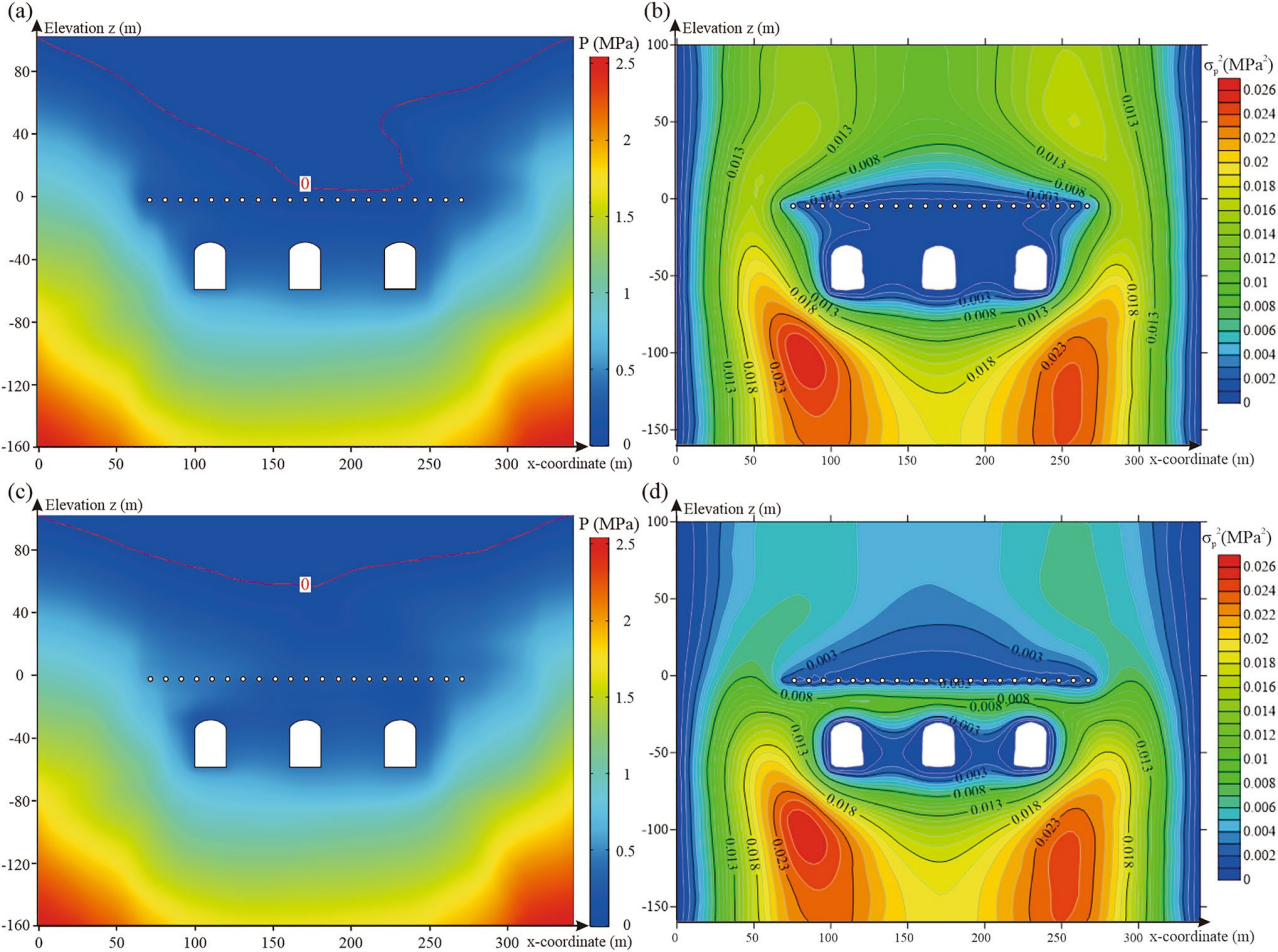
Characteristics distribution of pore water pressure with different water curtain borehole pressure: (**a**) pore water pressure distribution for one realisation of the random field under a water curtain borehole pressure of 0.1 MPa, (**b**) variance distribution of pore water pressure for 500 realisations of the random fields under a water curtain borehole pressure of 0.1 MPa, (**c**) pore water pressure distribution for one realisation of the random field under a water curtain borehole pressure of 0.5 MPa and (**d**) variance distribution of pore water pressure for 500 realisations of the random fields under a water curtain borehole pressure of 0.5 MPa. The figure was generated using the software COMSOL Multiphysics, version 5.3 (https://www.comsol.com/).

The 500 realisations of random fields were introduced into the numerical model of the underground water-sealed oil storage with different water curtain borehole pressures for seepage analysis. The pore water pressure distribution with different water curtain borehole pressures, for one realisation of the random field, is presented in Fig. 16a,c. With an increase in the pressure of the water curtain boreholes, the groundwater level above the caverns showed a clear upward trend. When the pressure of the water curtain boreholes was 0.5 MPa, the pore water pressure above the caverns and around the caverns increased significantly, and the water-sealed effect of the water curtain system gradually increased. The variance of pore water pressure with different water curtain borehole pressures for 500 realisations of the random fields was calculated and is shown in Fig. 16b,d. The results show that with an increase in the water curtain borehole pressure, the variance of the pore water pressure above the water curtain boreholes is significantly reduced, and the water pressure uncertainty is also reduced. We observed that the variance of the pore water pressure between the caverns and water curtain boreholes increased with a water curtain borehole pressure of 0.5 MPa. Thus, an excessively large water curtain borehole pressure increased the uncertainty of the water pressure above the caverns.

**Figure 17:**
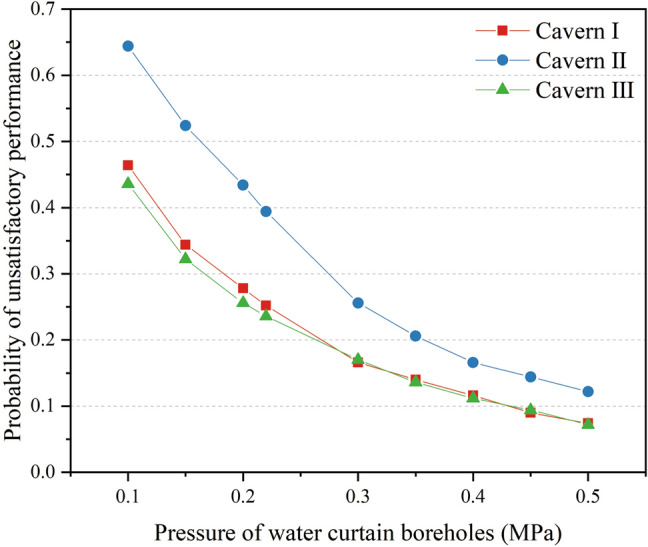
Probability of unsatisfactory performance with different water curtain borehole pressure.

For each value of the water curtain borehole pressure, the probability of unsatisfactory performance at the monitoring points at the top of the three caverns was calculated via MCS [Eqs. (6)–(8)]. The probability curves of unsatisfactory performance for water curtain borehole pressures ranging from 0.1 to 0.5 MPa are presented in Fig. 17. It was observed that the probability of unsatisfactory performance decreased nonlinearly with an increase in the water curtain borehole pressure. The probability curves for unsatisfactory performance were concave upward. The decline rate of the probability of unsatisfactory performance was greater at small water curtain borehole pressures, and decreased with increasing water curtain borehole pressure. Under a water curtain borehole pressure of 0.1 MPa, the probabilities of unsatisfactory performance for the three caverns were 0.464, 0.644, and 0.436, respectively. The probability of unsatisfactory water-sealed performance was high, and underground water-sealed oil storage posed a considerable leakage risk. When the water curtain borehole pressure increased from 0.1 to 0.3 MPa, the probability of unsatisfactory performance dropped sharply. The probability of unsatisfactory performance for the three caverns decreased by 0.298, 0.388, and 0.266, respectively, and the water-sealed effect of the water curtain system was effectively improved. However, when the water curtain borehole pressure was greater than 0.3 MPa, the decline rate of the probability of unsatisfactory performance gradually decreased. The results showed that an excessively large pressure of water curtain boreholes provided a small contribution to improving water curtain performance.

## Discussion

**Figure 18:**
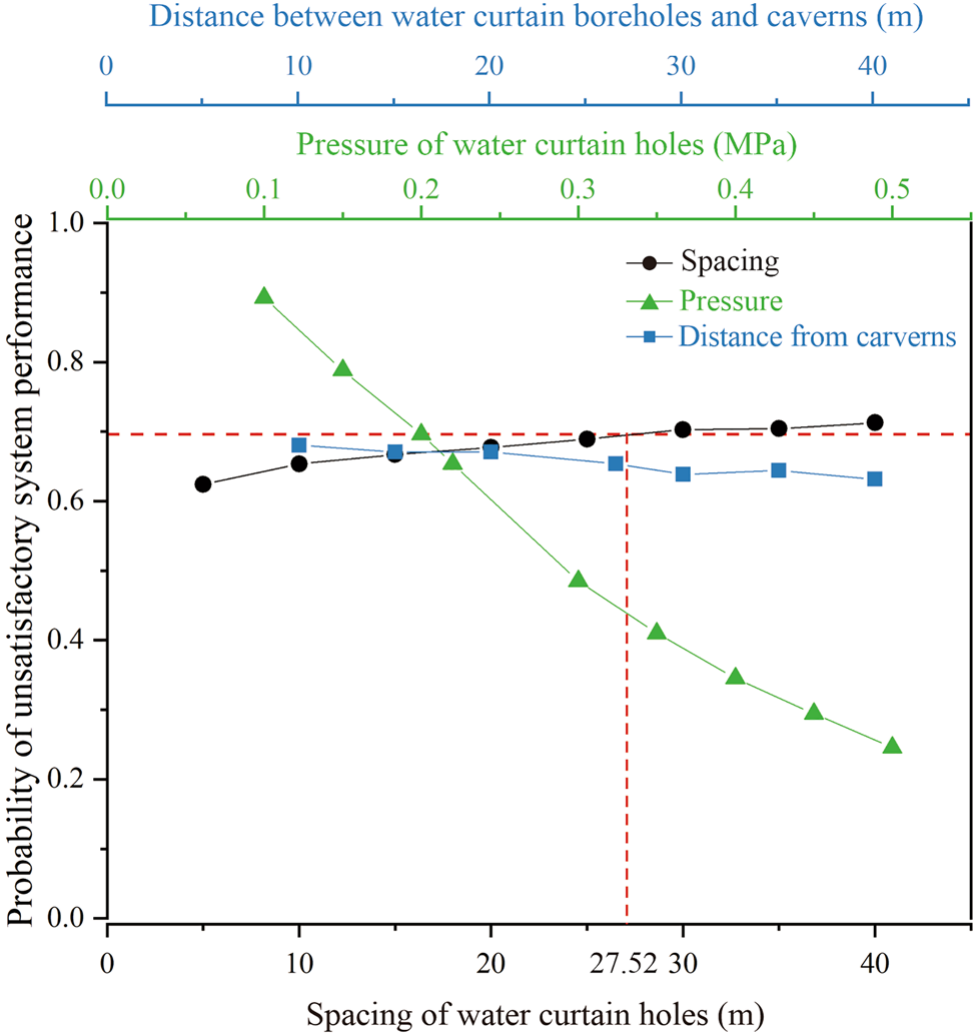
Probability of unsatisfactory system performance with different water curtain holes design parameters.

Underground water-sealed oil storage is regarded as a complex large-scale geological system for storing crude oil. During operation, unsatisfactory water-sealed performance of caverns threatens the normal operation of the entire underground water-sealed oil storage system. A comprehensive evaluation of an underground water-sealed oil storage system is required to optimise the design of the water curtain system. If underground water-sealed oil storage is treated as a series system, consisting of multiple caverns, the probability of satisfactory performance of the underground water-sealed oil storage system is the product of the probability of satisfactory performance of each cavern^41^. Based on the analysis results in “Influence of water curtain system design parameters on water-sealed reliability” section, the effects of different water curtain system schemes on the water-sealed reliability of the underground oil storage system were analysed. A safety water head of 20 m maintained in the water curtain system is recommended in the *Code for Design of Underground Storage in Rock Caverns* (2020)^42^. As shown in Fig. 18, when the water curtain borehole pressure is 0.22 MPa and the distance from the caverns is 26.5 m, the water curtain spacing of 27.52 m is the threshold to ensure the water-sealed safety in this case study. In addition, it should be noted that treating underground water-sealed oil storage as a series system will lead to relatively conservative probability results.

Based on the spatial statistical analysis of available dataset obtained from the borehole water injection test and water curtain boreholes injection fall-off test, random field theory was used in this study to reflect the spatial variability of the hydraulic conductivity of the surrounding rock mass at the site. However, because the borehole data was used without considering location, many realisations that did not reflect the hydraulic conductivity at the borehole locations were included in the random field simulation. In future research, the conditional simulation method can be applied to optimally utilise the available measurement data and to constrain the random fields^43^. This would reduce the uncertainty and lead to a more cost-effective design.

Moreover, the probability of unsatisfactory performance of underground water-sealed oil storage calculated in this study was the probability of oil leakage accident initiation, excluding the process from accident progression to oil leakage, whose probability is relatively small. The results of this study were limited to two-dimensional analysis. Further research is necessary to compare the effects of two-dimensional and three-dimensional approaches.

## Conclusions

Based on spatial statistical analysis of field data, this study combined random field theory and the finite element method within a Monte Carlo framework to analyse water curtain performance in underground oil storage considering the spatial variability of hydraulic conductivity. The following are the main conclusions of this study:

1. The distribution of the hydraulic conductivity of the surrounding rock at the site approximately obeyed the lognormal distribution, and the horizontal spatial correlation was greater than the vertical spatial correlation.
2. Ignoring the spatial variability of hydraulic conductivity may lead to an overestimation of the water-sealed reliability of underground oil storage. The difference between the horizontal spatial correlation and the vertical spatial correlation of the surrounding rock had a significant impact on the water-sealed effect of the water curtain system.
3. The decrease in the spacing of the water curtain boreholes and the increase in the water curtain holes pressure positively affected the water curtain performance. An excessively large pressure of water curtain boreholes made a small contribution to improving the water curtain performance. In addition, the distance between the water curtain holes and the caverns had the less significant affecting the water-sealed reliability of the storage cavern. This study can serve as a valuable reference for analogous engineering cases of underground water-sealed oil storage.

## Supplementary Information

Supplementary Information.

## Data Availability

All data generated or analysed during this study are included in this published article and its supplementary information files.
