# Supplementary material for: Probabilistic analysis of water-sealed performance in underground oil storage considering spatial variability of hydraulic conductivity
Source: Sci Rep. 2022 Aug 12;12:13782. doi: 10.1038/s41598-022-16960-3 (PMC9374700; doi:10.1038/s41598-022-16960-3)
Supplement: Supplementary file 1 — Supplementary Information. [file 41598_2022_16960_MOESM1_ESM.docx]

**Supplementary Information**

Tables 1 and 2 contain all the raw data in this study.

Table 1

Hydraulic conductivity data from eight boreholes water injection tests.

| Elevation  (m) | Hydraulic conductivity  (m/s) |  |  |  |  |  |  |  |
| --- | --- | --- | --- | --- | --- | --- | --- | --- |
|  | ZK1 | ZK3 | ZK6 | ZK13 | ZK14 | ZK18 | ZK24 | ZK31 |
| -20 | 1.15E-10 | 7.90E-08 | 6.13E-09 | 3.99E-08 | 1.12E-08 | 3.47E-09 | 3.37E-08 | 2.26E-08 |
| -30 | 2.54E-09 | 3.44E-08 | 4.80E-08 | 7.53E-08 | 4.18E-08 | 3.47E-09 | 1.62E-09 | 8.10E-09 |
| -40 | 9.25E-10 | 4.86E-08 | 1.73E-09 | 3.17E-08 | 3.43E-08 | 4.62E-09 | 4.61E-08 | 4.39E-09 |
| -50 | 2.31E-09 | 2.95E-08 | 2.31E-09 | 3.29E-08 | 1.12E-08 | 2.21E-08 | 6.48E-09 | 2.19E-09 |

Table 2

Hydraulic conductivity data from 784 water curtain boreholes injection fall-off tests.

| Horizontal distance  (m) | Hydraulic conductivity  (m/s) |  |  |  |  |  |  |  |  |  |
| --- | --- | --- | --- | --- | --- | --- | --- | --- | --- | --- |
|  | A-1 | A-2 | B-1 | B-2 | C-1 | C-2 | D-1 | D-2 | E-1 | E-2 |
| 10 | 1.21E-09 | -- | 1.06E-08 | 7.10E-08 | 4.53E-08 | 3.67E-08 | 8.51E-08 | 1.02E-07 | 3.80E-08 | 3.35E-09 |
| 20 | 1.21E-09 | -- | 1.68E-08 | 1.27E-07 | 3.67E-08 | 4.14E-08 | 9.45E-08 | 7.53E-08 | 7.13E-09 | 2.22E-08 |
| 30 | 1.21E-09 | 1.84E-09 | 1.69E-08 | 5.31E-08 | 4.14E-08 | 3.58E-08 | 1.01E-07 | 7.24E-08 | 9.60E-08 | 1.96E-08 |
| 40 | 1.21E-09 | 1.21E-09 | 7.21E-09 | 9.18E-08 | 3.58E-08 | 3.49E-08 | 7.18E-08 | 9.54E-08 | 8.23E-09 | 4.36E-09 |
| 50 | -- | 2.42E-09 | 2.65E-08 | 2.12E-08 | 3.49E-08 | 4.34E-08 | 9.05E-08 | 8.83E-08 | 7.13E-09 | 2.51E-09 |
| 60 | 7.76E-10 | -- | 1.73E-08 | 3.35E-10 | 4.34E-08 | 3.84E-08 | 1.07E-07 | 8.17E-08 | 3.85E-08 | 1.71E-08 |
| 70 | 1.29E-09 | -- | 1.17E-09 | 4.62E-08 | 3.84E-08 | 3.17E-08 | 8.50E-08 | 1.03E-07 | 2.82E-08 | 1.62E-08 |
| 80 | -- | 2.14E-08 | 1.17E-09 | 8.96E-08 | 3.17E-08 | 2.05E-08 | 8.70E-08 | 1.18E-07 | 4.22E-08 | 1.45E-08 |
| 90 | 3.18E-09 | 1.20E-07 | 4.69E-08 | 6.59E-08 | 2.05E-08 | 3.36E-08 | 8.05E-08 | 1.12E-07 | 2.33E-08 | 3.16E-08 |
| 100 | 1.45E-09 | 2.06E-08 | 2.01E-09 | 9.41E-08 | 3.36E-08 | 3.35E-08 | 1.73E-07 | 2.07E-07 | 1.69E-08 | 2.76E-08 |
| 110 | 1.69E-09 | 1.90E-08 | 1.34E-09 | 4.79E-08 | 3.35E-08 | 2.01E-08 | 8.49E-08 | 1.26E-07 | 2.51E-08 | 1.68E-08 |
| 120 | 5.36E-10 | 4.84E-09 | 3.35E-10 | 5.08E-08 | 2.01E-08 | 1.94E-08 | 1.37E-07 | 1.06E-07 | 5.48E-09 | 4.31E-08 |
| 130 | 4.83E-10 | 2.18E-09 | 2.18E-09 | 1.39E-08 | 1.94E-08 | 9.94E-08 | 1.27E-07 | 1.56E-07 | 9.51E-09 | 4.29E-08 |
| 140 | 4.83E-10 | 1.14E-08 | 3.35E-10 | 5.08E-08 | 9.94E-08 | 2.80E-08 | 1.22E-07 | 8.41E-08 | 7.13E-09 | 5.03E-08 |
| 150 | 5.29E-09 | 7.53E-09 | 3.72E-10 | 2.08E-08 | 2.80E-08 | 3.52E-08 | 1.08E-07 | 9.43E-08 | 3.83E-08 | 1.68E-08 |
| 160 | 5.16E-09 | 1.77E-08 | 2.16E-08 | 2.08E-08 | 3.52E-08 | 3.47E-08 | 1.14E-07 | 7.26E-08 | 2.92E-08 | 1.56E-08 |
| 170 | 4.23E-08 | 1.24E-08 | 5.34E-08 | 5.80E-08 | 3.47E-08 | 2.59E-08 | 4.18E-08 | 3.66E-08 | 3.96E-08 | 3.33E-08 |
| 180 | 1.36E-08 | 1.83E-08 | 1.08E-08 | 5.42E-08 | 2.59E-08 | 5.89E-08 | 4.84E-08 | 8.14E-08 | 3.24E-08 | 2.44E-08 |
| 190 | 2.19E-09 | 1.21E-08 | 2.44E-08 | 3.19E-08 | 5.89E-08 | 6.62E-08 | 3.00E-08 | 5.57E-08 | 2.73E-08 | 2.22E-08 |
| 200 | 2.56E-08 | 1.26E-08 | -- | 6.49E-08 | 6.62E-08 | 6.24E-08 | 9.05E-08 | 5.54E-08 | 3.37E-08 | 1.99E-08 |
| 210 | 1.69E-08 | 1.17E-08 | 3.52E-09 | 8.31E-08 | 6.24E-08 | 6.37E-08 | 4.12E-08 | 7.58E-08 | 3.50E-08 | 2.43E-08 |
| 220 | 3.91E-08 | 8.40E-09 | 1.84E-09 | 3.67E-08 | 6.37E-08 | 1.97E-08 | 4.22E-08 | 1.41E-07 | 2.66E-08 | 2.22E-08 |
| 230 | 3.91E-08 | 1.91E-08 | -- | 4.08E-08 | 1.97E-08 | 2.56E-08 | 4.02E-08 | 4.21E-08 | 3.58E-08 | 2.47E-08 |
| 240 | 2.05E-08 | 1.79E-08 | -- | 4.16E-08 | 2.56E-08 | 2.31E-08 | 6.01E-08 | 4.07E-08 | 1.57E-08 | 5.00E-08 |
| 250 | 3.05E-08 | 2.79E-08 | -- | 7.23E-08 | 2.31E-08 | 3.14E-08 | 1.11E-07 | 3.88E-08 | 5.97E-08 | 4.73E-08 |
| 260 | 4.44E-08 | 2.88E-08 | -- | 7.60E-08 | 3.14E-08 | 4.57E-08 | 1.07E-07 | 2.68E-08 | 3.32E-08 | 2.78E-08 |
| 270 | 1.66E-08 | 3.51E-08 | 4.55E-09 | 7.04E-08 | 4.57E-08 | 2.13E-08 | 3.35E-08 | 3.54E-08 | 4.21E-08 | 4.73E-08 |
| 280 | 2.58E-08 | 2.97E-08 | -- | 2.29E-08 | 2.13E-08 | 3.25E-08 | 5.67E-08 | 3.82E-08 | 5.40E-08 | 2.10E-08 |
| 290 | 2.04E-08 | 2.59E-08 | 3.02E-09 | 1.34E-09 | 3.25E-08 | 3.22E-08 | 5.49E-08 | 5.36E-08 | 3.69E-08 | 3.17E-08 |
| 300 | 2.15E-08 | 5.51E-08 | 2.45E-08 | 6.05E-08 | 3.22E-08 | 3.46E-08 | 6.91E-08 | 2.38E-08 | 3.15E-08 | 3.37E-08 |
| 310 | 2.85E-08 | 3.40E-08 | 1.99E-08 | 2.23E-09 | 3.46E-08 | 5.19E-08 | -- | -- | 1.19E-07 | 6.10E-08 |
| 320 | 2.78E-08 | 3.93E-08 | 1.14E-09 | 4.86E-08 | 5.19E-08 | 2.54E-08 | -- | 1.99E-08 | 4.08E-08 | 2.14E-08 |
| 330 | 3.14E-08 | 4.39E-08 | 7.09E-09 | 2.93E-09 | 2.54E-08 | 2.49E-08 | 1.68E-08 | 4.06E-08 | 3.63E-08 | 2.22E-08 |
| 340 | 4.16E-08 | 6.32E-08 | -- | 7.66E-09 | 2.49E-08 | 3.96E-08 | 3.96E-08 | 2.41E-08 | 3.15E-08 | 2.27E-08 |
| 350 | 2.68E-08 | 3.22E-08 | 1.27E-08 | 4.31E-09 | 3.96E-08 | 3.46E-08 | 1.73E-08 | 4.40E-08 | 2.42E-08 | 2.64E-08 |
| 360 | 3.61E-08 | 5.22E-08 | 7.75E-08 | 1.51E-09 | 3.46E-08 | 4.96E-08 | 1.71E-08 | 2.71E-08 | 3.23E-08 | 2.77E-08 |
| 370 | 3.07E-08 | 2.89E-08 | 2.01E-10 | 3.35E-10 | 4.96E-08 | 1.41E-08 | 1.53E-08 | 3.28E-08 | 2.99E-08 | 2.56E-08 |
| 380 | 2.06E-08 | 3.16E-08 | 6.84E-08 | 1.99E-08 | 1.41E-08 | 2.30E-08 | 2.56E-08 | 1.83E-08 | 4.28E-08 | 1.97E-08 |
| 390 | 2.68E-08 | 3.72E-08 | 2.03E-08 | 3.35E-10 | 2.30E-08 | 4.87E-08 | 2.51E-08 | 2.26E-08 | 3.19E-08 | 2.10E-08 |
| 400 | 2.66E-08 | 4.07E-08 | 2.98E-08 | 3.18E-08 | 4.87E-08 | 2.72E-08 | 2.24E-08 | 1.93E-08 | 2.85E-08 | 2.01E-08 |
| 410 | 2.95E-08 | 2.88E-08 | 1.99E-08 | 2.72E-08 | 2.72E-08 | 1.40E-08 | 2.51E-08 | 1.74E-08 | 3.19E-08 | 2.03E-08 |
| 420 | 4.10E-08 | 1.95E-08 | 1.83E-08 | 6.03E-09 | 1.40E-08 | 1.61E-08 | 1.83E-08 | 1.65E-08 | 3.49E-08 | 2.92E-08 |
| 430 | 2.62E-08 | 1.50E-08 | 1.12E-08 | 3.87E-08 | 1.61E-08 | 3.18E-08 | 2.06E-08 | 2.68E-08 | 3.22E-08 | 7.21E-08 |
| 440 | 2.47E-08 | 2.10E-08 | 8.38E-09 | 3.82E-08 | 3.18E-08 | 3.18E-08 | 1.94E-08 | 2.19E-08 | 3.18E-08 | 2.31E-08 |
| 450 | 2.02E-08 | 1.80E-08 | 4.08E-08 | 2.05E-08 | 3.18E-08 | 1.45E-08 | 7.13E-08 | 2.91E-08 | 3.03E-08 | 1.80E-08 |
| 460 | 1.19E-08 | 2.18E-08 | 3.69E-08 | -- | 1.45E-08 | 2.99E-08 | 9.05E-08 | 2.72E-08 | 8.22E-08 | 2.10E-08 |
| 470 | 1.80E-08 | 2.67E-08 | 2.23E-08 | 3.02E-08 | 2.99E-08 | 2.49E-08 | 8.38E-09 | 8.38E-09 | 2.44E-08 | 3.73E-08 |
| 480 | 2.36E-08 | 3.40E-08 | 3.15E-08 | 1.39E-08 | 2.49E-08 | 3.19E-08 | 3.35E-08 | 1.87E-08 | 3.84E-08 | 3.32E-08 |
| 490 | 4.36E-08 | 5.48E-08 | 3.32E-08 | 3.78E-08 | 3.19E-08 | 2.30E-08 | 1.51E-08 | 2.68E-08 | 2.92E-08 | 4.24E-08 |
| 500 | 1.28E-08 | 1.33E-08 | 1.28E-07 | 1.98E-08 | 2.30E-08 | 1.31E-08 | 2.23E-08 | 2.51E-08 | 3.38E-08 | 2.13E-08 |
| 510 | 1.73E-08 | 2.84E-08 | 9.11E-08 | 2.30E-08 | 1.31E-08 | 2.01E-08 | 2.19E-08 | 2.51E-08 | 3.87E-08 | 2.54E-08 |
| 520 | 3.93E-08 | 4.00E-08 | 7.74E-08 | 1.99E-08 | 2.01E-08 | 3.35E-09 | 4.78E-08 | 5.40E-08 | 3.99E-08 | 3.49E-08 |
| 530 | 2.24E-08 | 1.40E-08 | 6.45E-08 | 1.81E-08 | 3.35E-09 | 2.61E-08 | 5.49E-08 | 3.77E-08 | 3.36E-08 | 2.89E-08 |
| 540 | 4.22E-08 | 8.84E-09 | 5.28E-08 | 2.16E-08 | 2.61E-08 | 2.26E-08 | 2.74E-08 | 3.30E-08 | 2.94E-08 | 3.07E-08 |
| 550 | 5.63E-08 | 7.59E-09 | 6.37E-08 | 2.64E-08 | 2.26E-08 | 2.83E-08 | 4.60E-08 | 7.07E-08 | 2.68E-08 | 2.64E-08 |
| 560 | 5.97E-08 | 9.20E-09 | 6.20E-08 | 3.11E-08 | 2.83E-08 | 2.19E-08 | 4.78E-08 | 4.28E-08 | 4.88E-08 | 2.51E-08 |
| 570 | 1.91E-08 | 8.58E-09 | 6.87E-09 | 2.55E-08 | 2.19E-08 | 3.70E-08 | 3.02E-08 | 3.79E-08 | 2.04E-08 | 2.20E-08 |
| 580 | 1.44E-08 | 1.65E-08 | 1.16E-08 | 1.99E-08 | 3.70E-08 | 5.98E-08 | 8.28E-08 | 6.75E-08 | 5.82E-08 | 3.32E-08 |
| 590 | 2.64E-08 | 1.25E-08 | 2.15E-08 | 2.54E-08 | 5.98E-08 | 8.14E-08 | 7.71E-08 | 2.86E-08 | 2.39E-08 | 2.96E-08 |
| 600 | 4.33E-09 | 3.59E-09 | 3.35E-10 | 9.72E-09 | 8.14E-08 | 3.67E-08 | 4.90E-08 | 3.45E-08 | 2.42E-08 | 4.86E-08 |
| 610 | 1.82E-08 | 1.37E-08 | 8.05E-09 | 2.54E-08 | 3.67E-08 | 9.39E-08 | 3.24E-08 | 3.57E-08 | 1.74E-08 | 4.40E-08 |
| 620 | 1.72E-08 | 1.31E-08 | 1.49E-08 | 9.44E-08 | 9.39E-08 | 6.99E-08 | 2.81E-08 | 4.40E-08 | 2.88E-08 | 5.20E-08 |
| 630 | 5.14E-08 | 4.41E-09 | 7.31E-09 | 1.10E-07 | 6.99E-08 | 3.80E-08 | 2.39E-08 | 2.99E-08 | 4.79E-08 | 3.37E-08 |
| 640 | 1.28E-08 | 1.23E-08 | 1.33E-08 | 1.35E-07 | 3.80E-08 | 6.22E-08 | 3.14E-08 | 2.89E-08 | 2.54E-07 | -- |
| 650 | 5.75E-09 | 1.82E-08 | 8.55E-09 | 1.27E-07 | 6.22E-08 | 5.41E-08 | 2.82E-08 | 2.51E-08 | 2.38E-08 | -- |
| 660 | 1.07E-07 | 1.10E-08 | 3.04E-08 | 1.35E-07 | 5.41E-08 | 7.64E-08 | 2.80E-08 | 3.35E-08 | 1.53E-08 | 1.98E-09 |
| 670 | 7.77E-08 | 4.81E-09 | 8.62E-08 | 1.35E-07 | 7.64E-08 | 5.75E-08 | 3.58E-08 | 2.86E-08 | 2.75E-08 | -- |
| 680 | 1.01E-07 | 7.62E-09 | 1.57E-08 | 1.34E-07 | 5.75E-08 | 4.93E-08 | 2.84E-08 | 5.15E-08 | 1.02E-08 | -- |
| 690 | 1.75E-08 | 8.48E-09 | 2.04E-08 | 1.16E-07 | 4.93E-08 | 7.07E-08 | 2.50E-08 | 2.05E-08 | 1.15E-08 | -- |
| 700 | 1.93E-08 | 5.26E-09 | 4.76E-08 | 1.28E-07 | 7.07E-08 | 5.98E-08 | 2.56E-08 | 4.36E-08 | 2.94E-08 | -- |
| 710 | 1.13E-07 | 1.13E-08 | 4.11E-08 | 1.24E-07 | 5.98E-08 | 4.65E-08 | 3.52E-08 | 4.41E-08 | -- | -- |
| 720 | 1.17E-07 | 1.84E-08 | 7.42E-09 | 1.30E-07 | 4.65E-08 | 3.85E-08 | 7.20E-08 | 2.41E-08 | 3.71E-08 | -- |
| 730 | 1.18E-08 | 1.13E-08 | 8.55E-09 | 1.19E-07 | 3.85E-08 | 5.89E-08 | 6.49E-08 | 2.69E-08 | 8.91E-09 | -- |
| 740 | -- | 2.91E-08 | 1.08E-07 | 1.22E-07 | 5.89E-08 | 3.96E-08 | 5.33E-08 | 2.75E-08 | 1.81E-08 | 1.05E-08 |
| 750 | 2.08E-08 | 9.41E-09 | 8.05E-08 | 3.49E-08 | 3.96E-08 | 2.56E-08 | 3.14E-08 | 6.29E-08 | -- | -- |
| 760 | 1.05E-07 | 6.92E-09 | 1.56E-07 | -- | 2.56E-08 | -- | 4.13E-08 | -- | 4.55E-08 | 4.37E-08 |
| 770 | 4.19E-08 | -- | 1.91E-07 | -- | -- | -- | -- | -- | -- | -- |
| 780 | 1.99E-08 | -- | -- | -- | -- | -- | -- | -- | -- | 4.60E-08 |
| 790 | 1.13E-07 | -- | -- | -- | -- | -- | -- | -- | -- | -- |
